# Supplementary material for: A cross sectional survey of knowledge, attitude and practices related to the use of insecticides among farmers in industrial triangle of Punjab, Pakistan
Source: PLoS One. 2021 Aug 19;16(8):e0255454. doi: 10.1371/journal.pone.0255454 (PMC8376108; doi:10.1371/journal.pone.0255454)
Supplement: S1 Table — (PDF) [file pone.0255454.s001.pdf]

QUESTIONNAIRE

I am conducting this study towards completion of my Doctorate degree in Entomology. Please give me a few minutes of your time to complete this questionnaire. The questions that you will be asked include information on knowledge, attitude and practices regarding the application of pesticides and resistance development in ladybird beetle (*Coccinella septempunctata*). Please note that your contribution in this study is highly appreciated. The information you provide will be treated confidentially and with dignity. Answer the questions as honest as possible as that will allow us to have a clear understanding of what is known and unknown, and in turn, plan relevantly according to the needs of this community.

A. SOCIO DEMOGRAPHIC DETAILS.				
A1.	Gender<br>جنس	Male مرد		
		Female عورت		
A2.	Age<br>عمر			
A3.	Marital Status<br>ازدواجی حیثیت	Single<br>غیر شادی شدہ		
		Married<br>شادی شدہ		
		Divorced<br>طلاق یافتہ		
		Widowed<br>رنڈوا /بیوہ		
A4.	Education level<br>تعلیمی قابلیت.	No formal education<br>غیر رسمی تعلیم		
		Primary level<br>پرائمری لیول		
		Middle level<br>مڈل لیول		
		Secondary level<br>ثانوی لیول		
		Higher secondary level<br>اعلی ثانوی لیول		
		Under Graduate level<br>انڈر گریجویٹ		
		Graduate level<br>گریجویٹ لیول		
A5	Farm size (ha)<br>فارم کا رقبہ (ایکڑ میں)	< 0.1		
		0.11- 0.5		
		0.51-1.0		
		> 0.1		
A6	Irrigation method<br>آبپاشی کا نظام	Drip ڈرپ اریگیشن		
		Sprinkler<br>چھڑکاؤ کا نظام		
		Tube well<br>ٹیوب ویل		

		Canal Irrigation<br>نہری نظام		
		Other دیگر		
A7	Farming experience<br>کھیتی باڑی کا تجربہ			
A8	Working hours<br>کام کے اوقات			
B. AWARENESS AND KNOWLEDGE.				
B1	Which pesticides are most commonly used? (Name of product)<br>کونسی کیڑے مار دوا عام طور پر استعمال کرتے ہیں؟(نام لکھیں)			
B2	For which Crop Type pesticides are used?<br>کس قسم کی فصل کےلئے یہ کیڑے مار دوا استعمال کر رہے ہیں؟	Wheat گندم		
		Rice چاول		
		Sugar cane گنا		
		Millet جو		
		Sorghumجوار		
		Vegetables..سبزیاں		
B3	For which pest pesticides are used?<br>کس کیڑے کے لئے یہ کیڑے مار دوا استعمال کر رہے ہیں؟	Aphid<br>تیلا		
		Smut<br>جالے		
		Whitefly<br>سفید مکھی		
		Jassid<br>ٹڈے		
		Mildew<br>پھپھوندی		
		Mealy bug<br>سسری		
		Termites<br>دیمک		
B4	How many years have you been using pesticides?<br>کتنے سال سے یہ کیڑے مار دوا استعمال کر رہے ہیں؟			
B5	What is the Frequency of spray/ Ha used?<br>ایک ایکڑ میں کتنی کثرت سے استعمال کر رہے ہیں؟	Two times دو دفعہ		
		Three times تین دفعہ		
		Four times<br>چار دفعہ		
		Five Times پانچ دفعہ		
		Other.دیگر		
B6	Pesticides are Sprayed at which Stage of crop?<br>فصل کے کس درجہ پر کیڑے مار دوا استعمال کر رہے ہیں؟	Early stage<br>بیج بونے کے فورا بعد		
		Grown stage<br>اگنے کے بعد		
		Mature Stage		

		تیار ہونے پر		
		Harvesting stage<br>فصل کاٹتے وقت.		
B7	Which method is adopted for application of pesticides?<br>کونسا طریقہ استمعال میں لآیا جاتا ہے سپرے کرتے وقت؟	Aerial.ہوائی طریقہ		
		Mixture with water<br>پانی میں ملا کر.		
		Synergist<br>مدد گار		
B8	Is label information are followed during Pesticides handling?<br>جب کیڑے مار دوا لگا ئی جاتی ہے تو ہدایت میں دی گئی معلومات کو مدنظر رکھا جاتا ہے؟	Follow label Information<br>ہدایات میں دی گئی معلومات پر عمل کرتےہیں		
		Proper Dose<br>ادویات کی صحیح مقدار میں استعمال		
		Follow Schedule of spray<br>سپرے کے جدول پر عمل کیا جاتا ہے		
		Maintenance of Pesticide Container<br>ادویات کے ڈبوں کی حفاظت کا خیال رکھا جاتا ہے		
B9	Have you ever heard about alternative to synthetic pesticides?<br>کیا آپ نے کیڑے مار ادویات کے متبادل اشیاء کے بارے میں سنا ہے؟	Yes جی ہاں		
		No .جی نہیں		
B10	If yes then which of alternative to synthetic pesticides you heard?<br>اگر ہاں تو کونسے کیڑے مار ادویات کی متبادل اشیاء کے بارے میں سنا ہے؟	Bio pesticides<br>سند کوبی		
		Organic farming<br>نامیاتی کاشتکاری		
		Crop rotation<br>زرعی گردش		
		Cultivating crop mixtures<br>مختلف فصلوں کا مجموع کاشت کرنا.		
B11	Have you heard about IPM?<br>کیا **IPM** کے بارے میں آپ نے سنا ہے؟	Yes جی ہاں		
		No .جی نہیں		
B12	Have you ever tried IPM or biological control agents?<br>کیا آپ نے کبھی آئی پی ایم یا قدرتی کنٹرول کا استعمال کیا ہے ؟	Yes جی ہاں		
		No .جی نہیں		
B13	Do you have knowledge about natural enemies?<br>کیا آپ نے کبھی قدرتی دشمن حشرات کے بارے میں سنا ہے ؟	Yes جی ہاں		
		No .جی نہیں		
B14	Do you think pesticides are economical to	Yes جی ہاں		

	be used?<br>کیا آپ کے خیال میں کیڑے مار ادویات کا استعمال فائدہ مند ہے ؟	No .جی نہیں		
C. Farmer Practices				
C1	Crop Practices<br>کاشت کاری کے کونسے طریقہ کار کو استعمال کیا جاتا ہے ؟	Cultural Control<br>ثقافتی طریقہ		
		Chemical Control<br>کیمیائی طریقہ		
		Biological Control<br>بائیولوجیکال طریقہ		
C2		IPM<br>آئی پی ایم		
	Which methods of empty pesticide container disposal you use?<br>کونسے طریقے سے حالی کیڑے مار دوا کے ڈبے کو تلف کرتے ہیں ؟	Government collection<br>گورنمنٹ اکٹھا کرتے ہی۔		
		Burry<br>دفنا دیتےہیں		
		Burnt<br>جلا دیتے ہیں		
		Throw in trash<br>کوڑا دان میں پھینک دیتےہیں		
		Other<br>دیگر۔		
C3	Farmer Training And Skills<br>کسانوں کی تربیت اور مہارت ۔	Training About pesticides application.<br>کیڑے مار ادویات کے استعمال کے متعلق تربیت۔		
		Assistance from Agricultural Officer.<br>زرعی افسر سے رہنمائی لینا ۔		
		Ability to use acquired skills.<br>دی گئی مہارتوں کا استعمال۔		
C4	Which protective measures are adopted during spray?<br>کونسی احتیاطی تدابیر آپ سپرے کرتے ہوئے اپناتے ہیں ؟	Hand gloves.<br>دستانے		
		Eye glasses<br>چشمہ		
		Overall<br>حفاظتی لبادہ		
		Respirator<br>آلہ تنفس		
		Face Mask<br>فیس ماسک		
		Boot/Shoes<br>لمبے جوتے		
		Others.		

		دیگر		
**C5**	At which time of day you spray the pesticides? دن کے کس وقت ادویات کا سپرے کیا جاتا ہے ؟	Morning صبح		
		Noon دوپہر		
		Afternoon سہ پہر		
		Evening شام		

COMMENTS (If Any) رائے ۔

Thank you
