# Supplementary material for: A cross sectional survey of knowledge, attitude and practices related to the use of insecticides among farmers in industrial triangle of Punjab, Pakistan
Source: PLoS One. 2021 Aug 19;16(8):e0255454. doi: 10.1371/journal.pone.0255454 (PMC8376108; doi:10.1371/journal.pone.0255454)
Supplement: S2 Table — (PDF) [file pone.0255454.s002.pdf]

Supplementary data

Table 1

	**Category**	**Percentage**
**Gender**	Male	93.7
	Female	6.3
**Age**	10-20 years	9.7
	21-30 years	32.3
	31-40 years	35.0
	41-50 years	11.0
	51-60 years	9.0
	More Than 60 years	3.0
**Marital Status**	Single	11.0
	Married	85.0
	Divorced	2.7
	Widow/Widower	1.3
**Educational level**	Non Formal Education	34.3
	Primary Level	16.0
	Middle Level	8.3
	Secondary level	6.7
	Higher Secondary	11.0
	Under Graduate Level	11.3
	Graduate Level	12.3
**Form Size (ha)**	<0.1	28.7
	0.11-0.5	41.0
	0.51-1.0	16.0
	>0.1	14.0
**Irregation Method**	Drip	17.7
	Sprinkler	7.0
	Tube Well	72.0
	Canal Irregation	3.3
**Farming Experience**	1-3 years	18.0

	4-6 years	28.3
	7-9years	35.3
	10-12 years	13.0
	more than 12 years	5.0
**Working Hours daily**	1-3 hours	28.3
	4-6 hours	38.7
	7-9 hours	19.0
	10-12 hours	12.0
	more than 12 hours	2.0

Table 2

	**Category**	**Percentage**
**For which crop type pesticides are used?**	Wheat	31.0
	Rice	23.0
	Sugar Cane	1.0
	Millet	2.3
	Sorghum	4.0
	Vegetables	38.7
**Which Pesticides are most commonly used?**	Name not label	99.3
	Agrowar	.7
**For which Pest, Pesticides are used?**	Aphid	49.0
	Smut	10.0
	White Fly	12.0
	Jassid	13.7
	Mildew	10.3
	Mealy Bug	3.0
	Termites	2.0
**How many years have you been using pestisides?**	0-5 years	35.3
	6-10 years	39.0
	11-15 years	15.7
	More than 15 Years	10.0

**What is the frequency of spray/Ha used?**	Two times for a crop season	14.7
	Three times for a crop season	46.3
	Four times for a crop season	32.3
	Five times for a crop season	6.7
**Pesticides are sprayed at which stage of crop?**	Early Stage	67.3
	Grown Stage	18.7
	Mature Stage	8.3
	Harvesting Stage	5.7
**Pesticides are sprayed at which stage of crop?**	Areial	67.3
	Mixture with water	18.7
	Synergist	8.3

Table 3

**Is label information are followed during pesticides handling?**	Follow Label Information	45.7
	Proper Dose	22.3
	Follow Schedule of spray	14.7
	Maintenance of Pesticide Container	17.3
**If Yes then which of alternative to syenthatic pesticides you heard?**	Bio Pesticides	3.3
	Organic Farming	1.0
	Crop Rotation	88.3
	Cultivating Crop Mixture	7.3
**Crop Practices**	Cultural Control	27.0
	Chemical Control	60.3

	Biological Control	7.3
	IPM	5.3
**Which method of empty pesticide container disposal you use?**	Government Collection	3.7
	Burry	55.7
	Burnt	12.3
	Throw in Trash	28.3
**Farmer Training and Skills**	training about pestisides application	12.0
	Assistance from Agricultural Officer.	82.7
	Ability to use acquired skills	5.3
**Which protective measure are adopted during spray?**	Hand Gloves	53.3
	Eye Glasses	10.3
	Overall	1.7
	Respirator	1.7
	Face Mask	29.7
	Boot/Shoes	3.3
**At which time of day you spray the pesticides?**	Morning	54.7
	Noon	2.0
	AfterNoon	36.7
	Evening	6.7

Table 4

	**Category**	**Percentage**
**Have you ever heard about alternative to syenthatic pesticides?**	Yes	38.7
	No	61.3
**Have you heard about IPM?**	Yes	31.7
	No	68.3
**Have you ever tried IPM or biological control agents?**	Yes	29.3
	No	70.7
**Do you have knowledge of natural enimies?**	Yes	33.3
	No	66.7

**Do you think Pesticides are economical to be used?**	Yes	7.0
	No	93.0

Table 5

		Non Formal Education		Primary Level		Middle Level		Secondary level		Higher Secondary		Under Graduate Level		Graduate Level	
		P-value	OR (95%CI)	P-value	OR (95%CI)	P-value	OR (95%CI)	P-value	OR (95%CI)	P-value	OR (95%CI)	P-value	OR (95%CI)	P-value	OR (95%CI)
**Heard IPM**	**Yes**		1		1		1		1		1		1		1
	**No**	.000	2.29 (1.46-3.36)	.000	2.21 (1.46-3.36)	.011	2.20 (1.19-4.05)	.079	2.12 (.91-4.92)	.655	1.22 (.50-2.94)	.012	2.66 (1.24-5.73)	.009	2.77 (1.29-5.95)
**Biological Control**	**Yes**				1		1		1		1		1		1
	**No**	.000	2.43 (1.59-3.72)	.000	2.433 (1.59-3.72)	.011	2.200 (1.19-4.05)	.034	2.57 (1.07-6.15)	.187	1.85 (.74-4.65)	.061	2.00 (.97-4.12)	.004	3.25 (1.47-7.17)
**Natural Enemies**	**Yes**				1		1		1		1		1		1
	**No**	.025	1.57 (1.06-2.34)	.025	1.57 (1.06-2.34)	.011	2.20 (1.19-4.05)	.002	5.25 (1.80-15.29)	.374	1.50 (.61-3.67)	.002	3.71 (1.61-8.55)	.494	1.26 (.64-2.49)
**Follow label Information B8[a]**	**Follow**		1		1		1		1		1		1		1
	**Proper Dose**	.03	.59 (.36-.96)	.08	.52 (.25-1.08)	.06	.30 (.08-1.09)	.01	.15 (.03-.68)	.32	.66 (.30-1.48)	.12	.52 (.23-1.18)	.28	.35 (.13-.89)
	**Follow Schedule of spray**	.001	.40 (.23-.78)	.004	.23 (.09-.63)	.206	.50 (.17-1.46)	.014	.15 (.03-.68)	.011	.20 (.05-.69)	.016	.29 (.10-.79)	.028	.35 (.13-.89)
	**Maintenance of Pesticide Container**	.000	.34 (.19-.61)	.08	.52 (.25-1.08)	.46	.70 (.26-1.08)	.02	.23 (.06-.81)	.01	.26 (.08-.80)	.006	.17 (.05-.60)	.079	.47 (.20-1.09)
**Irrigation method**	**Drip**		1		1		1		1		1		1		1
	**Sprinkler**	.40	.42 (.18-.96)	.05	.22 (.04-1.02)	.	.	.	.	.73	1.25 (.33-4.65)	.142	.20 (.02-1.71)	.739	1.25 (.33-4.65)
	**Tube Well**	.000	3.78 (2.28-6.28)	.000	3.88 (1.86-8.09)	.006	4.0 (1.50-10.65)	.18	1.85 (.74-4.65)	.001	6.0 (2.08-17.29)	.001	5.20 (1.99-13.54)	.000	6.5 (2.26-18.62)
	**Canal Irrigation**	.005	.21 (.07-.61)	.054	.22 (.04-1.02)	.	.	.	.	.	.	.273	.40 (.07-2.06)	.423	.50 (.09-2.73)

Table 6

Table 7

		Non Formal Education		Primary Level		Middle Level		Secondary level		Higher Secondary		Under Graduate Level		Graduate Level	
		P-value	OR (95%CI)	P-value	OR (95%CI)	P-value	OR (95%CI)	P-value	OR (95%CI)	P-value	OR (95%CI)	P-value	OR (95%CI)	P-value	OR (95%CI)
**Crop Practices**	**Cultural Control**		1		1		1		1		1		1		1
	**Chemical Control**	.000	2.78 (1.72-4.48)	.090	1.73 (.918-3.27)	.39	1.44 (.61-3.37)	.166	2.00 (.75-5.32)	.006	3.228 (1.41-7.65)	.136	1.80 (.83-3.89)	.023	2.27 (1.11-4.61)
	**Biological Control**	.028	.43 (.20-.91)	.019	.26 (.08-.80)	.054	.22 (.04-1.02)	.	.	.118	.28 (.05-1.37)	.121	.40 (.12-1.27)	.	.
	**IPM**	.003	.26 (.10-.64)	.011	.20 (.05-.69)	.037	.11 (.01-.87)	.178	.33 (.06-1.65)	.069	.14 (.01-1.16)	.038	.20 (.04-.91)	.022	.09 (.01-.70)
**Disposal of empty containers**	**Government Collection**		1		1		1		1		1		1		1
	**Burry**	.000	63 (8.73-454-2)	.001	11 (2.58-46.7)	.054	4.5 (.97-20.8)	.080	4 (.84-18.8)	.005	18 (2.40-134.8)	.001	12 (2.83-50.7)	.002	23 (3.1-170.3)
	**Burnt**	.013	13 (1.70-99.3)	.423	2 (.36-10.9)	.423	2 (.36-10.9)	1	1 (.14-7.0)	.142	5 (.58-42.7)	.423	2 (.36-10.9)	.142	5 (.58-42.7)
	**Throw in Trash**	.001	26 (3.52-191.5)	.002	10 (2.3-42.7)	.038	5 (1.09-22.8)	.080	4 (.84-18.8)	.037	9 (1.14-71.03)	.423	2 (.36-10.91)	.050	8 (1-63.9)
**Training and Protective measures**	**Hand Gloves**		1		1		1		1		1		1		1
	**Eye Glasses**	.000	.17 (.09-.34)	.001	.16 (.05-.46)	.013	.07 (.01-.58)	.017	.08 (.01-.64)	.058	.40 (.15-1.03)	.002	.10 (.02-.42)	.034	.38 (.16-.93)
	**Overall**	.000	.01 (.002-.12)	.	.	.013	.07 (.01-.58)	.017	.08 (.01-.64)	.009	.06 (.009-.50)	.003	.05 (.007-.37)	.	.
	**Respirator**	.000	.03 (.009-.14)	.002	.04 (.005-.29)	.	.	.	.	.007	.13 (.03-.58)	.	.	.	.
	**Face Mask**	.004	.52 (.33-.81)	.163	.64 (.34-1.2)	.533	.76 (.33-1.75)	.057	.33 (.10-1.03)	.226	.60 (.26-1.3)	.074	.50 (.23-1.06)	.136	.55 (.25-1.20)
	**Boot/Shoes**	.000	.05 (.01-.16)	.001	.08 (.01-.33)	.	.	.019	.16 (.03-.74)	.	.	.003	.05 (.007-.37)	.003	.11 (.02-.47)

		Non Formal Education		Primary Level		Middle Level		Secondary level		Higher Secondary		Under Graduate Level		Graduate Level	
		P-value	OR (95%CI)	P-value	OR (95%CI)	P-value	OR (95%CI)	P-value	OR (95%CI)	P-value	OR (95%CI)	P-value	OR (95%CI)	P-value	OR (95%CI)
**Spray**	**TWO TIMES FOR A CROP SEASON**		1		1		1		1		1		1		1
	**THREE TIMES FOR A CROP SEASON**	.000	4.99 (2.56-9.38)	.024	2.71 (1.14-6.45)	.566	1.40 (.44-4.41)	.083	2.75 (.87-.8.63)	.069	2.26 (.92-7.29)	.068	2.28 (.94-.5.55)	.008	3.80 (1.41-10.1)
	**Four TIMES FOR A CROP SEASON**	.003	2.18 (1.41-5.60)	.048	2.42 (1.0-5.85)	.100	2.40 (.84-6.81)	1	1 (.250-3.99)	.069	2.60 (.92-7.29)	.469	1.42 (.54-3.75)	.206	2 (.68-5.85)
	**Five TIMES FOR A CROP SEASON**	.350	.63 (.24-1.64)	.566	.71 (.22-2.25)	.142	.20 (0.23-1.71)	.215	.25 (.02-2.23)	.273	.40 (.07-2.06)	.069	.143 (.01-1.16)	.484	.60 (.143-2.51)
