# Supplementary material for: A cross sectional survey of knowledge, attitude and practices related to the use of insecticides among farmers in industrial triangle of Punjab, Pakistan
Source: PLoS One. 2021 Aug 19;16(8):e0255454. doi: 10.1371/journal.pone.0255454 (PMC8376108; doi:10.1371/journal.pone.0255454)
Supplement: Attachment — Submitted filename: PONE-D-20-16862_reviewer.pdf [file pone.0255454.s004.pdf]

PLOS ONE

A Cross Sectional Survey of Knowledge, Attitude and Practices Related to the Use of Insecticides among Farmers in Industrial Triangle of Punjab, Pakistan

--Manuscript Draft--

**Manuscript Number:**	PONE-D-20-16862
**Article Type:**	Research Article
**Full Title:**	A Cross Sectional Survey of Knowledge, Attitude and Practices Related to the Use of Insecticides among Farmers in Industrial Triangle of Punjab, Pakistan
**Short Title:**	KAP Study
**Corresponding Author:**	Bakhtawer nasrullah<br>University of Gujrat<br>gujrat, punjab PAKISTAN
**Keywords:**	Insecticides<br>chemical control<br>Biological agents
**Abstract:**	The present study aimed to access the current knowledge, attitude and common practices of farmers about the use of insecticides against pest in industrial triangle of Punjab Pakistan between October and February 2020. A cross sectional study was conducted among farmers (n=300) in three locations of Punjab (Gujrat, Gujranwala and Sialkot). Farmers were interviewed using a questionnaire to collect data about the knowledge about biological control agents they have and at which level they utilize biological control method along with chemical control method. ANOVA was used to investigate potential predicators. The result shows almost all (93%) farmers were male and they did not know about the insecticides mode of action and its 2 chemical composition. They also do not have any knowledge about the biological control of pests. Farmers did not get any assistance or help from agriculture extension Officer. They even did not properly dispose of the empty containers of insecticides. Statistical analysis reveals that lack of education and awareness about biological control of pest depicts development of resistance and outbreak of secondary pest including health hazards and environmental pollution. Poor understanding about pests abundant use of insecticides, incorrect perception about application of insecticides and negligence regarding biological control show there is need to initiate public awareness programs to ensure the application of IPM and sustainable agriculture.
**Order of Authors:**	Bakhtawer nasrullah
	Sumera Afsheen
**Additional Information:**	
**Question**	**Response**
**Financial Disclosure**<br><br>Enter a financial disclosure statement that describes the sources of funding for the work included in this submission. Review the submission guidelines for detailed requirements. View published research articles from *PLOS ONE* for specific examples.<br><br>This statement is required for submission and **will appear in the published article** if the submission is accepted. Please make sure it is accurate.	The authors received no specific funding for this work.

Powered by Editorial Manager® and ProduXion Manager® from Aries Systems Corporation

**Unfunded studies**<br>Enter: *The author(s) received no specific funding for this work.*<br><br>**Funded studies**<br>Enter a statement with the following details:<br>• Initials of the authors who received each award<br>• Grant numbers awarded to each author<br>• The full name of each funder<br>• URL of each funder website<br>• Did the sponsors or funders play any role in the study design, data collection and analysis, decision to publish, or preparation of the manuscript?<br>• **NO** - Include this sentence at the end of your statement: *The funders had no role in study design, data collection and analysis, decision to publish, or preparation of the manuscript.*<br>• **YES** - Specify the role(s) played.<br><br>* typeset	
**Competing Interests**<br><br>Use the instructions below to enter a competing interest statement for this submission. On behalf of all authors, disclose any competing interests that could be perceived to bias this work—acknowledging all financial support and any other relevant financial or non-financial competing interests.<br><br>This statement **will appear in the published article** if the submission is accepted. Please make sure it is accurate. View published research articles from *PLOS ONE* for specific examples.	The authors have declared that no competing interests exist.

Powered by Editorial Manager® and ProduXion Manager® from Aries Systems Corporation

**NO authors have competing interests**<br><br>Enter: *The authors have declared that no competing interests exist.*<br><br>**Authors with competing interests**<br><br>Enter competing interest details beginning with this statement:<br><br>*I have read the journal's policy and the authors of this manuscript have the following competing interests: [insert competing interests here]*<br><br>* typeset	
**Ethics Statement**<br><br>Enter an ethics statement for this submission. This statement is required if the study involved:<br><br>• Human participants<br>• Human specimens or tissue<br>• Vertebrate animals or cephalopods<br>• Vertebrate embryos or tissues<br>• Field research<br><br>Write "N/A" if the submission does not require an ethics statement.<br><br>General guidance is provided below. Consult the submission guidelines for detailed instructions. **Make sure that all information entered here is included in the Methods section of the manuscript.**	Before interview main objectives were explained clearly to farmers later on by their willingness data were recorded on questionnaire by verbally asking the questions because<br>majority of farmers were not able to read and understand questionnaire. This study was approved<br>by all statuary forums of the University of Gujrat, Gujrat, Pakistan including the Departmental<br>review and research Committee (DRRC), Advanced study and research board (ASRB), Board of<br>faculty (BoF) and Academic Council.

Powered by Editorial Manager® and ProduXion Manager® from Aries Systems Corporation

**Format for specific study types**<br><br>**Human Subject Research (involving human participants and/or tissue)**<br>• Give the name of the institutional review board or ethics committee that approved the study<br>• Include the approval number and/or a statement indicating approval of this research<br>• Indicate the form of consent obtained (written/oral) or the reason that consent was not obtained (e.g. the data were analyzed anonymously)<br><br>**Animal Research (involving vertebrate animals, embryos or tissues)**<br>• Provide the name of the Institutional Animal Care and Use Committee (IACUC) or other relevant ethics board that reviewed the study protocol, and indicate whether they approved this research or granted a formal waiver of ethical approval<br>• Include an approval number if one was obtained<br>• If the study involved *non-human primates*, add *additional details* about animal welfare and steps taken to ameliorate suffering<br>• If anesthesia, euthanasia, or any kind of animal sacrifice is part of the study, include briefly which substances and/or methods were applied<br><br>**Field Research**<br><br>Include the following details if this study involves the collection of plant, animal, or other materials from a natural setting:<br>• Field permit number<br>• Name of the institution or relevant body that granted permission	
**Data Availability**<br><br>Authors are required to make all data underlying the findings described fully available, without restriction, and from the time of publication. PLOS allows rare exceptions to address legal and ethical concerns. See the PLOS Data Policy and FAQ for detailed information.	No - some restrictions will apply

Powered by Editorial Manager® and ProduXion Manager® from Aries Systems Corporation

A Data Availability Statement describing where the data can be found is required at submission. Your answers to this question constitute the Data Availability Statement and **will be published in the article**, if accepted.<br><br>**Important:** Stating 'data available on request from the author' is not sufficient. If your data are only available upon request, select 'No' for the first question and explain your exceptional situation in the text box.<br><br>Do the authors confirm that all data underlying the findings described in their manuscript are fully available without restriction?	
**Describe where the data may be found in full sentences. If you are copying our sample text, replace any instances of XXX with the appropriate details.**<br><br>• If the data are **held or will be held in a public repository**, include URLs, accession numbers or DOIs. If this information will only be available after acceptance, indicate this by ticking the box below. For example: *All XXX files are available from the XXX database (accession number(s) XXX, XXX.).*<br>• If the data are all contained **within the manuscript and/or Supporting Information files**, enter the following: *All relevant data are within the manuscript and its Supporting Information files.*<br>• If neither of these applies but you are able to provide **details of access elsewhere**, with or without limitations, please do so. For example:<br><br>*Data cannot be shared publicly because of [XXX]. Data are available from the XXX Institutional Data Access / Ethics Committee (contact via XXX) for researchers who meet the criteria for access to confidential data.*<br><br>*The data underlying the results presented in the study are available from (include the name of the third party*	Data cannot be shared publicly because this data is confidential.It is for the completion of PhD Degree.

and contact information or URL).<br>• This text is appropriate if the data are owned by a third party and authors do not have permission to share the data.<br><br>* typeset	
Additional data availability information:	Tick here if your circumstances are not covered by the questions above and you need the journal’s help to make your data available.

Powered by Editorial Manager® and ProduXion Manager® from Aries Systems Corporation

Manuscript

Click here to access/download;Manuscript;A Cross Sectional clean copy.docx

A Cross Sectional Survey of Knowledge, Attitude and Practices Related to the Use of Insecticides among Farmers in Industrial Triangle of Punjab, Pakistan

Bakhtawer, Sumera Afsheen

Department of Zoology, University of Gujrat, Hafiz Hayat Campus, Jalalpur Jattan Road, Gujrat, Punjab 50700, Pakistan.

E-mail: sumera.afsheen@uog.edu.pk

Abstract

The present study was aimed to access the current knowledge, attitude and common practices of farmers about the use of insecticides against pest in industrial triangle of Province Punjab, Pakistan. This study was conducted during October and December, 2019. In this study farmers (n=300) took part from three localities of Province, Punjab (Gujrat, Gujranwala and Sialkot). Farmers were interviewed using a questionnaire to collect data about the knowledge of pest control by use of chemical method, biological method and combination of both as well as utilization of predators to eradicate the pests. ANOVA was used to investigate potential predicators. The result shows almost all (93%) farmers were male and they did not know about the insecticides mode of action and its chemical composition. They also do not have any knowledge about the biological control of pests. Farmers did not get any assistance or help from Agriculture Extension Officer. They even did not properly dispose off the empty containers of insecticides. Statistical analysis reveals that lack of education and awareness about biological

control of pest depicts development of resistance and outbreak of secondary pest including health hazards and environmental pollution.

Poor understanding about pests, abundant use of insecticides, incorrect perception about application of insecticides and negligence regarding biological control shows there is need to initiate public awareness programs to ensure the application of IPM and sustainable agriculture.

Introduction

Chemical control of pests in agriculture sector is the major prominent method to get higher yield of crops. Various chemicals including Organophosphate, Neonicotinoids, Carbamates and Pyrethoids are abundantly used. Integration of chemical control with biological control is the basic part of IPM but unfortunately it has been rarely applied [1].

It has been observed that farmers mostly rely on chemicals to protect their crops from pest's attacks without knowing the harmful consequences of insecticides. This practice leads not only to development of resistance in pests but also reduce the number of useful insects, outbreak of secondary pests, health issues and environmental pollution [2].

In Province Punjab variety of crops are cultivated including wheat, Rice, Cotton, Sugarcane, fruits and vegetables. In these crops field sap sucking insects cause damage to crops at all stages of crop development. Farmers of this region rely on insecticidal control of pests [3]. These farmers did not have any awareness about the chemical nature of insecticides [4]. They also do not have any assistance or training from agricultural extension sector regarding application of insecticides [5]. For sustainable agriculture there is need to reduce the use of insecticides and

control the pest attack. It is very necessary to adopt the biological control method along with chemical control in order to bring sustainability. For this purpose public awareness is very important to give general knowledge to farmers. Now they can use predators to control the pest. Use of natural predators can be economical to farmers as well as it is ecofriendly.

Material and Method

This study was conducted industrial triangle of Punjab (Gujrat, Gujranwala and Sialkot) Pakistan.

Study area

A cross sectional study was conducted during OCT-DEC 2019 by using questionnaire based interview of farmers. This survey was carried out in industrial triangle including district Gujrat, Gujranwala and Sialkot Fig 01. These districts were selected on the basis of operational convenience and industrial importance of this region. The demographic characteristics of this region are given in Table 1.

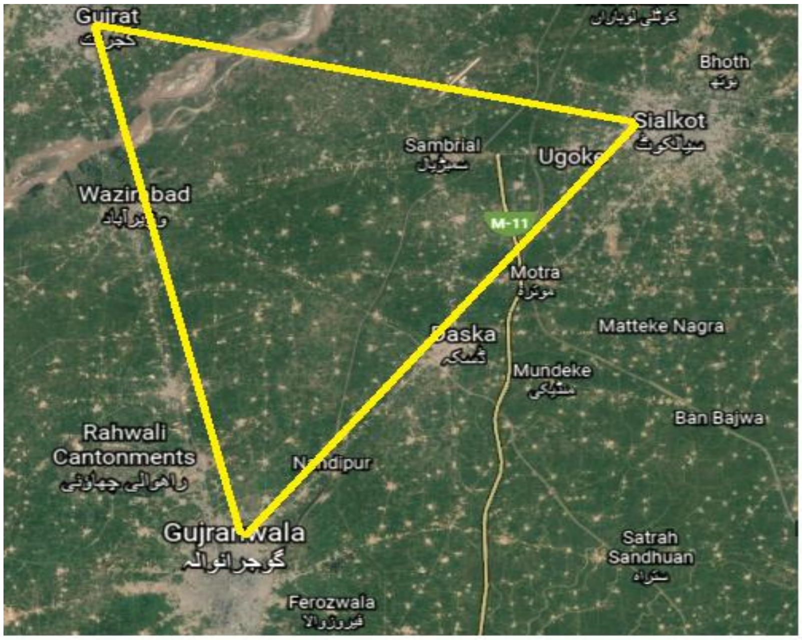

Map indicating current study area (Map created by using Google Maps).

Province	District	Area<br><br>Sq km	Population		
			Number	Rural %	Urban %
Punjab	Gujranwala	3622	5014196	41.18	58.81
	Gujrat	3192	2756110	96.98	30.02
	Sialkot	3016	3893672	70.64	29.36

Table 1: Demographic characteristic of study area source: Pakistan bureau of statistics 2017.

In first step questionnaire was prepared and survey was conducted from a sub sample of 20 Farmers who were reluctant to participate in study. On the basis of results of pretest this survey was further modified to reduce the deficiencies felt at that time. In second step a comprehensive

survey was conducted among 300 farmers of District Gujrat, Gujranwala and Sialkot of Punjab province.

A structured questionnaire having open ended and close ended questions was used to collect information by using Urdu and Punjabi Language. The questionnaire consist up of three sections, First part is related to sociodemographic characters of farmers. Second part related to perceptions and third related to the attitude and practices of farmers.

Interview procedure

Each village was visited for 4-5 days and 10-15 farmers were interviewed per day. Before interview main objectives of research were explained to them and later on voluntary they involved giving information.

Ethics Statement

Before interview main objectives were explained clearly to farmers. Later on by their willingness data was recorded on questionnaire by verbally asking the questions because majority of farmers were not able to read and understand questionnaire. This study was approved by all statuary forums of the University of Gujrat, Gujrat, Pakistan including the Departmental Review and Research Committee (DRRC), Advanced Study and Research Board (ASRB), Board of Faculty (BoF) and Academic Council.

Results

A total of 300 farmers from three Districts were participated in the study.

Demographic characteristics of Respondents

The majority of farmers were male (93.66 %) and 34% had no formal education. 11.33% **of** respondents had education level up to higher secondary school and only 12.33% Graduated. Most of the farmers were in 31-40 (34 %) year’s age. Detail of demographic characteristics of farmers is given in Table 2.

Table 2: Sociodemographic characteristics of farmers in three localities of province Punjab Pakistan (n= 300)

Gender	Character	Number	% age
	Male	281	93.66
	Female	19	6.33
Age	10-20	30	10
	21-30	99	33
	31-40	102	34
	41-50	33	11
	51-60	27	9
	More than 60	9	3
Marital status	Single	33	11
	Married	255	85
	Divorced/ widowed	12	4
Education level	Illiterate	102	34

	Primary level	48	16
	Middle level	25	8.33
	Secondary level	20	6.66
	Higher secondary level	34	11.33
	Under graduate level	34	11.33
	Graduate level	37	12.33

Knowledge of respondents

Most of the farmers have 1 hector area for the cultivation of crops. This data indicate that these farmers had small farming units. Most common irrigation method adopted by the farmers was tube well. 7% farmer used sprinkle method while only 3% utilized canal system in the region for irrigation.

38 % farmers spend 4-6 hours for farming activities daily. Only 2% reported to do work more than 12 hours. Most of the respondents had 7-9 years of farming experience. (Table 3)

Table 3: knowledge of farmers in three localities of province Punjab Pakistan (n= 300)

**Characters**		**Number**	**% age**
Farm size (ha)	Less than 1	87	29
	1-1/2	123	41

	2	48	16
	More than 2	42	14
Irrigation method	Drip method	54	18
	Sprinkle	21	7
	Tube well	216	72
	Canal system	9	3
Farming experience	1-3 years	54	18
	4-6 years	87	29
	7-9 years	105	35
	10-12 years	39	13
	More than 12	15	5
Working hours	1-3 hours	87	29
	4-6 hours	114	38
	7-9 hours	57	19
	10-12 hours	36	12
	More than 12 hours	6	2

Knowledge of respondents regarding pesticides

Most of the insecticides used by the farmers were belong to Acetamprid, Cypermethrim, Lambda Cyhalothrin and Dimethoate group. Detail information about insecticide is given in Table 4.

Table 4: Knowledge of farmers in three localities of province Punjab Pakistan (n= 300)			
Knowledge of farmers			
Pesticides used	Acetamprid<br>Cypermethrin<br>Lamda cyhalothrin<br>Dimethoate		
Characters		Number	% age
Crops	Wheat	93	31
	Rice	69	23
	Sugarcane	3	1
	Millet	6	2
	Sorghum	12	4
	Vegetables	117	39
Pests	Sumuts	30	10

	Aphids<br><br>Whiteflies<br><br>Jassids<br><br>Mildew<br><br>Mealybugs<br><br>Termites	147<br><br>36<br><br>42<br><br>30<br><br>9<br><br>6	49<br><br>12<br><br>14<br><br>10<br><br>3<br><br>2
Frequency of spray	Two time<br><br>Three time<br><br>Four time<br><br>Five times	44<br><br>139<br><br>97<br><br>20	14.66<br><br>46.33<br><br>32.33<br><br>6.66
Stage of crop	Early stage<br><br>Grown stage<br><br>Mature stage<br><br>Harvesting stage	202<br><br>56<br><br>25<br><br>17	67.33<br><br>18.66<br><br>8.33<br><br>5.66
Mode of application	Arial<br><br>Mix with water<br><br>Synergist	214<br><br>40<br><br>46	71.33<br><br>13.33<br><br>15.33
Lebal instructions	Follow	126	42

	Proper dose	66	22
	Schedule	45	15
	Maintain containers	63	21
Alternative synthetic insecticides	Yes	111	37
	No	189	63
In case yes	Crop rotation	3.33	3
	Organic farming	1.11	1
	Crop rotation	96.57	87
	Crop mixtures	7.77	7
Knowledge about IPM	Yes	94	31.33
	No	206	68.66
Biological control	Yes	104	34.66
	No	196	65.33
Knowledge about natural enemies	Yes	99	33
	No	201	67

The insecticides were used during the cultivation of different crops include wheat, rice, sugarcane, millet, Sorghum and vegetables. Highest proportion of these insecticides 39% were

used for vegetables followed by wheat 31% and Rice 23% .Major pest reported for these crops were smuts, Aphids, whiteflies, Jessed, mildew, mealy bugs and termites. Out of which 49% of insecticides were used to control aphids. These insecticides were sprayed three (46.33%) to four (32.33%) times by majority of farmers. These sprays were used during the early stage of pest growth (67.33%). Only 5.66% were reported to use spray at harvesting stage of pests. 71.33% of farmer sprays their crops by Arial method, 15.33% used synergist and only 13.33% used mixture with water. Label instruction for preparing dose is very crucial step to properly use insecticides against pests. 42% of respondents know to understand the label instruction during preparation for spray. 22% of respondents were able to made proper dose. 15% of the respondents follow the schedule for spray and 21% were founded to maintain empty containers of insecticides.

When the farmers were asked about the alternative synthetic insecticides, 63 % responded No. 68.6% did not have knowledge about IPM. Most of the respondent 65.3% did not know about biological control method of pests.

Common practices of farmers

Control of pest by the use of chemicals 55.33% was common practice followed by the cultural control methods (32.33%). Only a small fraction of respondents have adopted IPM 5.33% and biological control method 7 % in their farming routines. These farmers 50.33 % burry the empty containers and 14% of respondents burnt them while 31% throw them in trash without any processing. As well as precautionary measures are concerned, hand gloves 44% and facemask 41% or covering the face with cloth are most adopted precautionary measures. use of glasses, overall, respirator and long shoes were mostly neglected measures among farmers during the use of insecticides. For the proper use of insecticides, training is very important but

unfortunately 7% of respondents have any acquired skill and 12% have training about the use of insecticides while most of them get name of products and use them for pests just by asking from agriculture officer.

Table 5: Common practices of farmers in three localities of province Punjab Pakistan (n= 300)			
	Character	Number	%age
Crop practices methods	Cultural	97	32.33
	Chemical	166	55.33
	Biological	21	7
	IPM	16	5.33
Practices for Disposal of insecticides containers	Govt collection	11	3.66
	Burry	151	50.33
	Burnt	43	14.33
	Throw in trash	95	31.66
Farmer training /skill	Pesticides application	36	12
	Agriculture officer guidance	243	80
	Use acquired skills	21	7
Practice to use Protective	Hand gloves	132	44

measures	Glasses<br><br>Overall<br><br>Respirator<br><br>Face mask<br><br>Boot/shoes	33<br><br>3<br><br>3<br><br>123<br><br>6	11<br><br>1<br><br>1<br><br>41<br><br>2
Time of spray	Morning<br><br>Noon<br><br>After noon<br><br>Evening	132<br><br>6<br><br>111<br><br>21	44<br><br>2<br><br>37<br><br>7

Univarient regression model showed farmers having non formal education **are at** high level of risk as compared to the respondents that know about the mode of action of insecticides. There was strong correlation between education level and common practices of farmers. However knowledge about the IPM and biological control method of pests was very low indicated the need of awareness campaign among farmers. Chemical control was the dominated method practiced among the farmers for the control of pests.

Discussion

More than half of respondents adopted chemical method to control the pest infestation but the knowledge about the accurate rate of pest infestation was very poor. Use of insecticide is recommended only when pest infestation reaches to the threshold level while this study reveals respondents did not know about the actual pest infestation rate as well as they did not receive any training or skill about insecticide usage. They also did not use all preventing measures during the application of insecticides. Sankol *et al* 2016 also reported that 71% of farmers did not use safety measures. The education level also plays an important role. Low education level of farmers increases the risk factors in fields[6].

Data reveals most of respondents had no trainings from extension officer about the use of insecticides as Aldose *et al* (2018) 79.5% of farmers did not received any training whereas 12.3%, 5.1% and 3.1% received 2,4,and more than four trainings respectively. Similarly according to Aslam *et al* (2007) farmers of Pakistan had low level of knowledge about insecticides use however training by extension officer can be an effective step to reduce risk factors[7]. According to another study conducted in Ethiopia by Negatu *et al* (2016) 85% of the farmers had no any training regarding application of insecticides [8], while Ibitayo (2006) reported similar trend of farmers [4]. According to this study 98% of farmers did not had any training about insecticides application. In UK a study conducted regarding risk assessment about safe use of insecticides among workers. It indicated that proper training and adoption of proper safety measures can reduce the health hazards [9].

Alternative pest control method is very important component of IPM and very essential for sustainability. The result of current study shows 63% of respondents did not had any idea about

alternative methods. Among 37% that know about alternative method crop rotation only practiced among farmers.

Biological control is a very effective method along with the chemical control but unfortunately due to low level of knowledge and awareness 65.3% of respondents did not know about this. This unawareness leads to force them only rely on insecticidal control method.

Conclusion

The findings of this survey demonstrate poor understanding and risky practices adopted by the farmers are the major contributing factors towards resistance development and health issues. For underdeveloped countries like Pakistan a comprehensive and well planned program targeting on alternative pest control method and use of biological agents along with insecticides need to be initiated that can reduce the total dependency on chemicals.

There is a die need to start up the farmer's awareness campaigns as well as need to organize farmers by giving them trainings about proper disposal of empty containers to minimize the resistance development and resurgence of secondary pests.

Acknowledgement

The author sincerely thanks to the farmers and extension agriculture department for participating and contributing to this study.

References

1. Rodrigues S R A, spindola F A, Torres B J, Herbert A A S, colares F. response of different populations of seven lady beetle species to Lamda-Cyhalothrin with record of resistance. Ecotoxicology and environmental safety 2013; 96 , 53-60.
2. Abbas N, Shad S A , Ismail M, resistance to conventional and new insecticides in houseflies (diptera: muscidae) from poultry facilities in Punjab pakistan. J. Econ. Entomol.2014; 108: 826-833
3. Basit M, Saeed S, saleem MA ,Denholm I, Shah M. Detection of resistance , cross resistance and stability of resistance to new chemistry insecticides in helicoverpa armigera. J Econ Entomol 2013; 106:1414-1422.
4. Ibitayo OO, Egyptian farmers,attitude and behavior regarding aricuktural pesticides: implications for pesticides risk communication. Risk anal 2006; 26(4) 989-995.
5. Sodavy p, sitha M, Nugent r, Murphy h. situation analysis: farmers awareness and perceptions of the effects of pesticides on their health .field document.combodia :FAO Community IPM PROGRAM .2000
6. Sankoh AI, Whittle R, Semple KT, Jones KC, Sweetman AJ(2016) An assessment of the impacts of pesticide use on theenvironment and health of rice farmers in Sierra Leone.Environment International 94: 458-466.
7. Aslam M, Ali T, Zafar MI, Ahmad M (2007) Training needs offruit growers regarding pesticide use for sustainableenvironmental health in Punjab. Pakistan Journal of AgriculturalSciences 44: 511-518.
8. Negatu B, Kromhout H, Mekonnen Y, Vermeulen R (2016) Useof Chemical Pesticides in Ethiopia: A Cross-SectionalComparative Study on Knowledge Attitude and Practice ofFarmers and Farm Workers in Three Farming Systems. AnnualOccupation Hygiene 16

9. Remoundou K, Brennan M, Hart A, Frewer LJ (2014) PesticideRisk Perceptions, Knowledge, and Attitudes of Operators,Workers, and Residents: A Review of the Literature. Human and Ecological Risk Assessment 20:1113 –1138

Click here to access/download
Supporting Information
ethical approval.docx

Click here to access/download
Supporting Information
bakht paper submitted to plos one highlited.docx
