# Supplementary material for: A cross sectional survey of knowledge, attitude and practices related to the use of insecticides among farmers in industrial triangle of Punjab, Pakistan
Source: PLoS One. 2021 Aug 19;16(8):e0255454. doi: 10.1371/journal.pone.0255454 (PMC8376108; doi:10.1371/journal.pone.0255454)
Supplement: Attachment — Submitted filename: PONE-D-20-16862_R1_reviewer.pdf [file pone.0255454.s006.pdf]

PLOS ONE

A Cross Sectional Survey of Knowledge, Attitude and Practices Related to the Use of Insecticides among Farmers in Industrial Triangle of Punjab, Pakistan

--Manuscript Draft--

**Manuscript Number:**	PONE-D-20-16862R1
**Article Type:**	Research Article
**Full Title:**	A Cross Sectional Survey of Knowledge, Attitude and Practices Related to the Use of Insecticides among Farmers in Industrial Triangle of Punjab, Pakistan
**Short Title:**	KAP Study
**Corresponding Author:**	Bakhtawer nasrullah<br>University of Gujrat<br>gujrat, punjab PAKISTAN
**Keywords:**	Insecticides<br>chemical control<br>Biological agents
**Abstract:**	The present study aimed to access the current knowledge, attitude and common practices of farmers about the use of insecticides against pest in industrial triangle of Punjab Pakistan between October and February 2020. A cross sectional study was conducted among farmers (n=300) in three locations of Punjab (Gujrat, Gujranwala and Sialkot). Farmers were interviewed using a questionnaire to collect data about the knowledge about biological control agents they have and at which level they utilize biological control method along with chemical control method. ANOVA was used to investigate potential predicators. The result shows almost all (93%) farmers were male and they did not know about the insecticides mode of action and its 2 chemical composition. They also do not have any knowledge about the biological control of pests. Farmers did not get any assistance or help from agriculture extension Officer. They even did not properly dispose of the empty containers of insecticides. Statistical analysis reveals that lack of education and awareness about biological control of pest depicts development of resistance and outbreak of secondary pest including health hazards and environmental pollution. Poor understanding about pests abundant use of insecticides, incorrect perception about application of insecticides and negligence regarding biological control show there is need to initiate public awareness programs to ensure the application of IPM and sustainable agriculture.
**Order of Authors:**	Bakhtawer nasrullah
	Sumera Afsheen
**Response to Reviewers:**	file attached.
**Additional Information:**	
**Question**	**Response**
**Financial Disclosure**<br><br>Enter a financial disclosure statement that describes the sources of funding for the work included in this submission. Review the submission guidelines for detailed requirements. View published research articles from *PLOS ONE* for specific examples.<br><br>This statement is required for submission and **will appear in the published article** if the submission is accepted. Please make	The authors received no specific funding for this work.

Powered by Editorial Manager® and ProduXion Manager® from Aries Systems Corporation

sure it is accurate.<br><br>**Unfunded studies**<br>Enter: *The author(s) received no specific funding for this work.*<br><br>**Funded studies**<br>Enter a statement with the following details:<br>• Initials of the authors who received each award<br>• Grant numbers awarded to each author<br>• The full name of each funder<br>• URL of each funder website<br>• Did the sponsors or funders play any role in the study design, data collection and analysis, decision to publish, or preparation of the manuscript?<br>• **NO** - Include this sentence at the end of your statement: *The funders had no role in study design, data collection and analysis, decision to publish, or preparation of the manuscript.*<br>• **YES** - Specify the role(s) played.<br><br>* typeset	
**Competing Interests**<br><br>Use the instructions below to enter a competing interest statement for this submission. On behalf of all authors, disclose any competing interests that could be perceived to bias this work—acknowledging all financial support and any other relevant financial or non-financial competing interests.<br><br>This statement **will appear in the published article** if the submission is accepted. Please make sure it is accurate. View published research articles from *PLOS ONE* for specific examples.	The authors have declared that no competing interests exist.

**NO authors have competing interests**<br><br>Enter: *The authors have declared that no competing interests exist.*<br><br>**Authors with competing interests**<br><br>Enter competing interest details beginning with this statement:<br><br>*I have read the journal's policy and the authors of this manuscript have the following competing interests: [insert competing interests here]*<br><br>* typeset	
**Ethics Statement**<br><br>Enter an ethics statement for this submission. This statement is required if the study involved:<br><br>• Human participants<br>• Human specimens or tissue<br>• Vertebrate animals or cephalopods<br>• Vertebrate embryos or tissues<br>• Field research<br><br>Write "N/A" if the submission does not require an ethics statement.<br><br>General guidance is provided below. Consult the submission guidelines for detailed instructions. **Make sure that all information entered here is included in the Methods section of the manuscript.**	Before interview main objectives were explained clearly to farmers later on by their willingness data were recorded on questionnaire by verbally asking the questions because<br>majority of farmers were not able to read and understand questionnaire. This study was approved<br>by all statuary forums of the University of Gujrat, Gujrat, Pakistan including the Departmental<br>review and research Committee (DRRC), Advanced study and research board (ASRB), Board of<br>faculty (BoF) and Academic Council.

**Format for specific study types**<br><br>**Human Subject Research (involving human participants and/or tissue)**<br>• Give the name of the institutional review board or ethics committee that approved the study<br>• Include the approval number and/or a statement indicating approval of this research<br>• Indicate the form of consent obtained (written/oral) or the reason that consent was not obtained (e.g. the data were analyzed anonymously)<br><br>**Animal Research (involving vertebrate animals, embryos or tissues)**<br>• Provide the name of the Institutional Animal Care and Use Committee (IACUC) or other relevant ethics board that reviewed the study protocol, and indicate whether they approved this research or granted a formal waiver of ethical approval<br>• Include an approval number if one was obtained<br>• If the study involved *non-human primates*, add *additional details* about animal welfare and steps taken to ameliorate suffering<br>• If anesthesia, euthanasia, or any kind of animal sacrifice is part of the study, include briefly which substances and/or methods were applied<br><br>**Field Research**<br><br>Include the following details if this study involves the collection of plant, animal, or other materials from a natural setting:<br>• Field permit number<br>• Name of the institution or relevant body that granted permission	
**Data Availability**<br><br>Authors are required to make all data underlying the findings described fully available, without restriction, and from the time of publication. PLOS allows rare exceptions to address legal and ethical concerns. See the PLOS Data Policy and FAQ for detailed information.	Yes - all data are fully available without restriction

A Data Availability Statement describing where the data can be found is required at submission. Your answers to this question constitute the Data Availability Statement and **will be published in the article**, if accepted.<br><br>**Important:** Stating 'data available on request from the author' is not sufficient. If your data are only available upon request, select 'No' for the first question and explain your exceptional situation in the text box.<br><br>Do the authors confirm that all data underlying the findings described in their manuscript are fully available without restriction?	
**Describe where the data may be found in full sentences. If you are copying our sample text, replace any instances of XXX with the appropriate details.**<br><br>• If the data are **held or will be held in a public repository**, include URLs, accession numbers or DOIs. If this information will only be available after acceptance, indicate this by ticking the box below. For example: *All XXX files are available from the XXX database (accession number(s) XXX, XXX.*).<br>• If the data are all contained **within the manuscript and/or Supporting Information files**, enter the following: *All relevant data are within the manuscript and its Supporting Information files.*<br>• If neither of these applies but you are able to provide **details of access elsewhere**, with or without limitations, please do so. For example:<br><br>*Data cannot be shared publicly because of [XXX]. Data are available from the XXX Institutional Data Access / Ethics Committee (contact via XXX) for researchers who meet the criteria for access to confidential data.*<br><br>*The data underlying the results presented in the study are available from (include the name of the third party*	Data is attached.

and contact information or URL).<br>• This text is appropriate if the data are owned by a third party and authors do not have permission to share the data.<br><br>* typeset	
Additional data availability information:	Tick here if your circumstances are not covered by the questions above and you need the journal’s help to make your data available.

Powered by Editorial Manager® and ProduXion Manager® from Aries Systems Corporation

Manuscript

Click here to access/download;Manuscript;clean copy of manuscript.docx

A Cross Sectional Survey of Knowledge, Attitude and Practices Related to the Use of Insecticides among Farmers in Industrial Triangle of Punjab, Pakistan

Bakhtawer, Sumera Afsheen

Department of Zoology, University of Gujrat, Hafiz Hayat Campus, Jalalpur Jattan Road, Gujrat, Punjab 50700, Pakistan.

E-mail: sumera.afsheen@uog.edu.pk

Abstract

Pesticides in Pakistan are abundantly utilized for pest control in agriculture sector. The over and unsafe of insecticides plus poor handling, leads to the development of resistance, outbreak of secondary pests and hazardous impact on environment. The present study was aimed to access the current knowledge, attitude and common practices of farmers about the use of insecticides against pest in industrial triangle of Province Punjab, Pakistan. This study was conducted during October and December, 2019. In this study farmers (n=300) took part from three localities of Province, Punjab (Gujrat, Gujranwala and Sialkot). Farmers were interviewed using a questionnaire to collect data about the knowledge of pest control by use of chemical method, biological method and combination of both as well as utilization of predators to eradicate the pests. The result shows almost all (93%) farmers were male and they did not know about the insecticides mode of action and its chemical composition. They also do not have any knowledge about the biological control of pests. Farmers did not get any assistance or help from Agriculture Extension Officer. They even did not properly dispose off the empty containers of insecticides. Statistical analysis reveals that lack of education and awareness about biological control of pest depicts development of resistance and outbreak of secondary pest including health hazards and environmental pollution. Poor understanding about pests, abundant use of insecticides, incorrect perception about application of insecticides and negligence regarding biological control shows there is need to initiate public awareness programs to ensure the application of IPM and sustainable agriculture.

Introduction

Being an agricultural country economy of Pakistan based on agriculture sector. 1.8 billion people round the globe deal with pesticides to increase the yield of crops and protect them from pest attack. 42.5 percent people in rural areas of Pakistan involved in agricultural activities. Chemical control of pests in agriculture sector is the major prominent method to maximize productivity of crops.

Various chemicals including Organophosphate, Neonicotinoids, Carbamates and Pyrethroids are abundantly used. 108 types of insecticides, 39 types of herbicides 6 types of rodenticides and 5 types of aracicides were reported to be used in Pakistan for different crops. [illegible] use of pesticides have increased 9[illegible]% or more per hac[illegible]er in most developing countries including Pakistan.

It has been observed that farmers mostly rely on chemicals to protect their crops from pest's attacks without knowing the harmful consequences of insecticides. This practice leads not only to development of resistance in pests but also reduce the number of useful insects, outbreak of secondary pests, health issues and environmental pollution [2].

In Province Punjab variety of crops are cultivated including wheat, Rice, Cotton, Sugarcane, fruits and vegetables. In these crops field sap sucking insects cause damage to crops at all stages of crop development. Farmers of this region rely on insecticidal control of pests [3]. These farmers did not have any awareness about the chemical nature of insecticides [4]. They also do not have any assistance or training from agricultural extension sector regarding application of insecticides [5]. Due to lack of knowledge about harmful results of pesticides farmers did not pay attention about taking precautionary measures. Improper disposal of Empty containers that

contain surplus enough quantity of pesticides leads to the pollution as well as resistance development in non target species.

For sustainable agriculture there is need to reduce the use of insecticides and control the pest attack by other means. It is very necessary to adopt the biological control method along with chemical control in order to bring sustainability. For this purpose public awareness is very important to give general knowledge to farmers about the use of pesticides. Integration of chemical control with biological control is the basic part of IPM but unfortunately it has been rarely applied [1]. Use of natural predators can be economical to farmers as well as it is ecofriendly.

By keeping in view all the factors current study was designed to access the knowledge of farmers about pesticides application and alternative methods to control pests. This will help the agriculture department to design a comprehensive plan to educate farmers as well as built up an effective IPM strategy to control pests attack, secure food and bring sustainability.

Material and Method

Study area

A cross sectional study was conducted during October 2019 to February 2020 by using questionnaire based interview of farmers. In this survey face to face conversation was done with farmers. Firstly in local language aim of study was described later on after their willingness questions were asked verbally and their response was noted on questionnaire. This survey was carried out in industrial triangle including Gujrat, Gujranwala and Sialkot. These sites were selected on the basis of operational convenience and industrial importance of this region. Due to

availability of water and suitability of climatic conditions various crops like wheat, rice, fruits and vegetables cultivated in this region. Farmers of this region use insecticides abundantly to protect their crops from pests. The demographic characteristics of this region are given in Table 1.

**Province**	**District**	**Area Sq km**	**Population**		
			**Number**	**Rural %**	**Urban %**
Punjab	Gujranwala	3622	5014196	41.18	58.82
	Gujrat	3192	2756110	96.98	03.02
	Sialkot	3016	3893672	70.64	29.36

Table 1: Demographic characteristic of study area source: Pakistan bureau of statistics 2017.

From each site six localities were further selected randomly. From Gujrat six localities include Jalal pur jattan (JPJ), Karianwala (KN), Dolat Nagar (DN), Wazirabad (w), Lalamusa (LM) and Kharian (KH). From Gujranwala site ferozawala (FW), Uggochak (UG), Veropal Chatha (VC), Ali pur Chatha (AC), Qila miyan singh (QS) and Kot Ladha (KL) were selected for data collection. Daska (D), sambrial (S1), Sahowal (S2), Majra Kalan (MK), Dera sanda (SD) and Sahowala (S3) from Sialkot site were included in survey study. Detail of study area represented in Map 01.

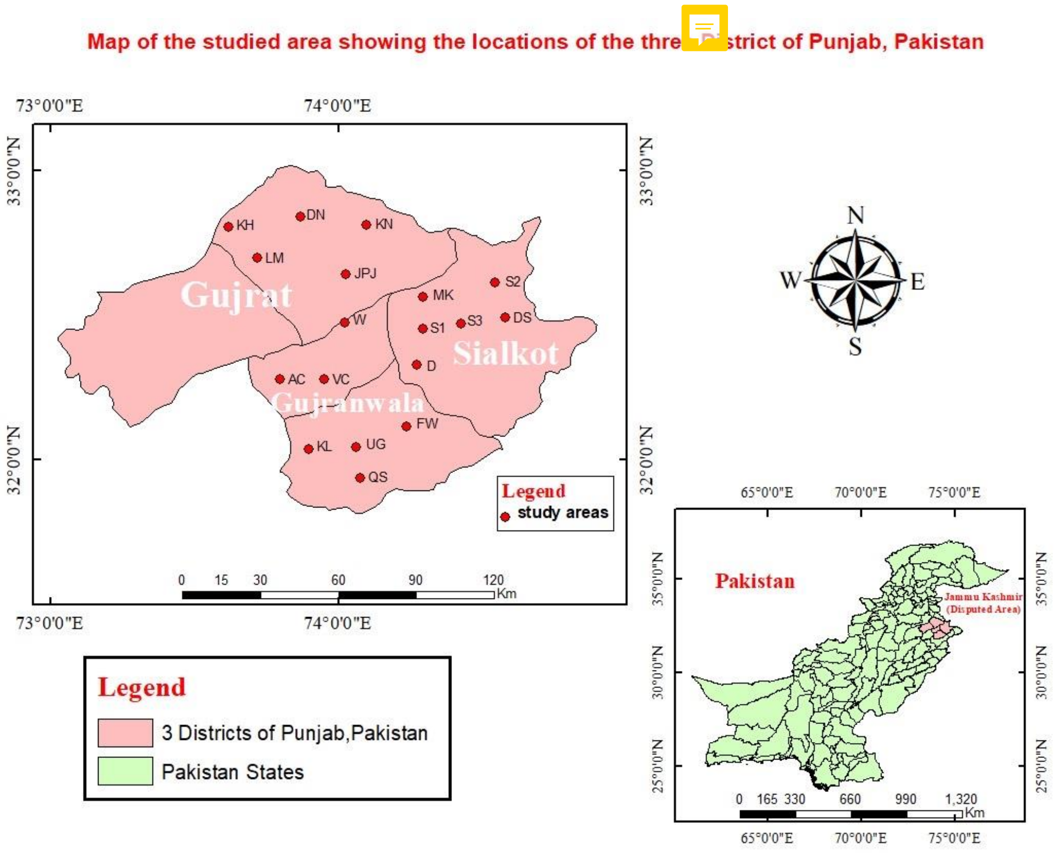

Ma[illegible]dicating current study area (Map created by using GIS-ARc).

In first step questionnaire was prepared and survey was conducted from a sub [illegible]mple of 20 [illegible]ners who were reluctant to participate in study. On the basis of results of pretest this survey was further modified to reduce the deficiencies felt at that time. In second step a comprehensive survey was conducted among 300 farmers of District Gujrat, Gujranwala and Sialkot of Punjab province.

Interview procedure

Each village was visited for 4-5 days and 10-15 on average farmers were interviewed per day via face to face conversatio[illegible]efore interview main objectives were explained clearly to

farmers. Later on by their willingness data was recorded on questionnaire by verbally asking the questions because majority of farmers were not able to read and understand questionnaire.

A structured questionnaire having open ended and close ended questions was used to collect information by using Urdu and Punjabi Language. The questionnaire consis[illegible] of three sections, First part is related to sociodemographic characters of farmers such as gender, marital status, Age, Education level , Farm area, irrigation method, farming Experience and working hours they spend in their crops were determined. Second part related to perceptions like which insecticide is better and they prefer to use more, which crop and which pest most frequently targeted, how they prepare the spray dose for targeted pests, they have any idea about alternate pest control method like IPM, Biological agent or natural enemies and third part related to the attitude and practices of farmers like how they protect themselves during spray, they use protective equipment or not and they acquired any skill or training from any department or agency.

Ethics Statement

This study was approved by all statuary forums of the University of Gujrat, Gujrat, Pakistan including the Departmental Review and Research Committee (DRRC), Advanced Study and Research Board (ASRB), Board of Faculty (BoF) and Academic Council.

Results

A total of 300 farmers from three Districts were participated in the study. Random sampling was done. Most of the farmers were involved in cultivation of wheat, rice, millet, sorghum and vegetables in this region. The response rate was 100% farmers did not refuse to participate.

Demographic characteristics of Respondents

The majority of farmers were male (93.66 %) while only 5.4% women were involved in farming. Education is a very important variable for the assessment of farmer's knowledge and practices. It was founded that 34% farmers were illiterate, 16% had primary level, 6% had middle level education, 11.33% of respondents had education level up to higher secondary school and only 12.33% were graduated. Figure 01(a) represents the detail of the education level of farmers. Age group of the farmers ranged from 20-60 years with an average of 31-40 (34 %) years. Detail of age in years was represented in figure 01 (b).

Knowledge of respondents

Most of the farmers have 1 hector area for the cultivation of crops. This data indicate that these farmers had small farming units. Most common irrigation method adopted by the farmers was tube well (72%) followed by the drip method (18%) while 3% was reported to utilized the canal system for irrigation purpose.

38 % farmers spend 4-6 hours for farming activities daily. Only 2% reported to do work more than 12 hours. Most of the respondents had 7-9 years of farming experience. Detail of farm area, irrigation method, Farming Experience and Working hours is given in Table 2.

Table 2: Knowledge of farmers in three localities of province Punjab Pakistan (n= 300)

**Characters**		**Number**	**Percentage**
Farm Area (ha)	Less than 1	87	29
	1-1/2	123	41
	2	48	16
	More than 2	42	14

Irrigation method	Drip method	54	18
	Sprinkle	21	7
	Tube well	216	72
	Canal system	9	3
Farming experience	1-3 years	54	18
	4-6 years	87	29
	7-9 years	105	35
	10-12 years	39	13
	More than 12	15	5
Working hours	1-3 hours	87	29
	4-6 hours	114	38
	7-9 hours	57	19
	10-12 hours	36	12
	More than 12 hours	6	2

Knowledge of respondents regarding pesticides

Majority of farmers deals with Acetamiprid, Cypermethrim, Lambda Cyhalothrin and Dimethoate belongs to the organophosphate and pyrethoids group of insecticides (WHO category II). Pest infestation was reported by the farmers include smuts, Aphids, Whiteflies, jassids, mildew, mealybugs and termites in this area. For the control of pest attack 46% farmers used to spray their crops three times, 32% sprayed Four times and 6% were reported to spray their crops five times. These sprays were used during the early stage of pest growth (67.33%). Only 5.66% were reported to use spray at harvesting stage of pests. 71.33% of farmer sprays their crops by Aerial method, 15.33% used synergist and only 13.33% used mixture with water. Label instruction for preparing dose is very crucial step to properly use insecticides against pests. 42% of respondents

understand the label instruction during preparation for spray. 22% of respondents were able to made proper dose. 15% of the respondents follow the schedule for spray and 21% were founded to maintain empty containers of insecticides. When the farmers were asked about the alternative synthetic insecticides, 63 % responded No while 37% responded Yes. 68.6% did not have knowledge about IPM. Most of the respondent 65.3% did not know about biological control method of pests. Detail information about knowledge of farmers about insecticides usage and alternative pest control methods is given in Table 3.

Table 3: Knowledge of farmers in three localities of province Punjab Pakistan (n= 300)

~~Knowledge of farmers~~				
Insecticides used		Acetamprid		
		Cypermethrin		
		Lambda cyhalothrin		
		Dimethoate		
Characters			Number	Percentage
Crops		Wheat	93	31
		Rice	69	23
		Sugarcane	3	1
		Millet	6	2
		Sorghum	12	4
		Vegetables	117	39
Pests	Scientific name	Common name		
	Ustilaginaceae	Sumuts	30	10
	Aphidoidea	Aphids	147	49
	Aleyrodidae	Whiteflies	36	12
	Amrasca biguttula	Jassids	42	14
	Golovinomyces orontii	Mildew	30	10

Pseudococcidae	Mealybugs	9	3
Isoptera	Termites	6	2
Frequency of spray (per Month)	Two time	44	14.66
	Three time	139	46.33
	Four time	97	32.33
	Five times	20	6.66
Stage of crop	Early stage	202	67.33
	Grown stage	56	18.67
	Mature stage	25	8.33
	Harvesting stage	17	5.67
Mode of application	Arial	214	71.33
	Mix with water	40	13.33
	Synergist	46	15.33
Lebal instructions	Follow	126	42
	Proper dose	66	22
	Schedule	45	15
	Maintain containers	63	21
Alternative synthetic insecticides	Yes	111	37
	No	189	63
In case yes	Crop rotation	3.33	3
	Organic farming	1.11	1
	Crop rotation	96.57	87
	Crop mixtures	7.77	7
Knowledge about IPM	Yes	94	31.33
	No	206	68.67
Biological control	Yes	104	34.67
	No	196	65.33
Knowledge about natural enemies	Yes	99	33
	No	201	67

Common practices of farmers

Chemical control of pest (55.33%) was common practice followed by the cultural control methods (32.33%) Cultural control refers to the manipulation of old practices like date management with respect to pest outbreak etc. Only a small fraction of respondents have adopted IPM (5.33%) and biological control method (7 %) in their farming routines. 50.33 % farmers burry the empty containers and 14% of respondents burnt them while 31.67% throw them in trash without any processing while 3.67% of farmers go for Government collection centers for disposal of empty containers of insecticides. As well as precautionary measures are concerned, hand gloves 44% and facemask 41% or covering the face with cloth are most adopted precautionary measures. Use of glasses, overall, respirator and long shoes were mostly neglected measures among farmers during the use of insecticides. For the proper use of insecticides, training is very important but unfortunately 7% of respondents have any acquired skill and 12% have training about the use of insecticides while most of them get name of products and use them for pests just by asking from agriculture officer. Detail of common practices of farmers is given in Table 4.

Table 4: Common practices of farmers in three localities of province Punjab Pakistan (n= 300)

	Character	Number	Percentage
Crop practices methods	Cultural	97	32.33
	Chemical	166	55.33
	Biological	21	7
	IPM	16	5.33

Practices for Disposal of insecticides containers	Govt collection	11	3.67
	Burry	151	50.33
	Burnt	43	14.33
	Throw in trash	95	31.67
Farmer training /skill	Pesticides application	36	12
	Agriculture officer guidance	243	80
	Use acquired skills	21	7
Practice to use Protective measures	Hand gloves	132	44
	Glasses	33	11
	Overall	3	1
	Respirator	3	1
	Face mask	123	41
	Boot/shoes	6	2
Time of spray	Morning	132	44
	Noon	16	5.33
	After noon	111	37
	Evening	41	13.67

Analysis revealed farmers having non formal education are at high level of risk as compared to the respondents that know about the mode of action of insecticides. There was strong correlation between education level and common practices of farmers. There was strong association between knowledge and label instructions given on products for proper dose preparation by farmers similarly time of spray, frequency of spray, use of protective equipment and disposal of empty insecticides containers has strong association with the knowledge of farmers. However knowledge about the IPM and biological control method of pests was very low indicated the need of awareness campaign among farmers. Chemical control was the dominated method practiced among the farmers for the control of pests.

The univariate regression analysis showed that all factors had significant differences with the education level of farmers ($p < 0.001$). Table 5 shows the knowledge of farmers about the alternative methods like IPM, Biological control and natural enemies is very poor however the farmers having knowledge about them are more likely to reduce the harmful impact of insecticides.

Table 05: Summary of univariate regression analysis column of the table refers to the education level of the farmers and rows refer the practices of farmers. Shown are the odd Ration (OR) and 95% confidence interval (95% CI) .

		P-value	OR (95%CI)	P-value	OR (95%CI)	P-value	OR (95%CI)	P-value	OR (95%CI)	P-value	OR (95%CI)	P-value	OR (95%CI)	P-value	OR (95%CI)
**Heard IPM**	**Yes**		1		1		1		1		1		1		1
	**No**	.000	2.29 (1.46-3.36)	.000	2.21 (1.46-3.36)	.011	2.20 (1.19-4.05)	.079	2.12 (.91-4.92)	.655	1.22 (.50-2.94)	.012	2.66 (1.24-5.73)	.009	2.77 (1.29-5.95)
**Biological Control**	**Yes**				1		1		1		1		1		1
	**No**	.000	2.43 (1.59-3.72)	.000	2.433 (1.59-3.72)	.011	2.200 (1.19-4.05)	.034	2.57 (1.07-6.15)	.187	1.85 (.74-4.65)	.061	2.00 (.97-4.12)	.004	3.25 (1.47-7.17)
**Natural Enemies**	**Yes**				1		1		1		1		1		1
	**No**	.025	1.57 (1.06-2.34)	.025	1.57 (1.06-2.34)	.011	2.20 (1.19-4.05)	.002	5.25 (1.80-15.29)	.374	1.50 (.61-3.67)	.002	3.71 (1.61-8.55)	.494	1.26 (.64-2.49)
**Follow label Information B8[a]**	**Follow**		1		1		1		1		1		1		1
	**Proper Dose**	.03	.59 (.36-.96)	.08	.52 (.25-1.08)	.06	.30 (.08-1.09)	.01	.15 (.03-.68)	.32	.66 (.30-1.48)	.12	.52 (.23-1.18)	.28	.35 (.13-.89)
	**Follow Schedule of spray**	.001	.40 (.23-.78)	.004	.23 (.09-.63)	.206	.50 (.17-1.46)	.014	.15 (.03-.68)	.011	.20 (.05-.69)	.016	.29 (.10-.79)	.028	.35 (.13-.89)
	**Maintenance of Pesticide Container**	.000	.34 (.19-.61)	.08	.52 (.25-1.08)	.46	.70 (.26-1.08)	.02	.23 (.06-.81)	.01	.26 (.08-.80)	.006	.17 (.05-.60)	.079	.47 (.20-1.09)
**Irrigation method**	**Drip**		1		1		1		1		1		1		1
	**Sprinkler**	.40	.42 (.18-.96)	.05	.22 (.04-1.02)	.	.	.	.	.73	1.25 (.33-4.65)	.142	.20 (.02-1.71)	.739	1.25 (.33-4.65)
	**Tube Well**	.000	3.78 (2.28-6.28)	.000	3.88 (1.86-8.09)	.006	4.0 (1.50-10.65)	.18	1.85 (.74-4.65)	.001	6.0 (2.08-17.29)	.001	5.20 (1.99-13.54)	.000	6.5 (2.26-18.62)
	**Canal Irrigation**	.005	.21 (.07-.61)	.054	.22 (.04-1.02)	.	.	.	.	.	.	.273	.40 (.07-2.06)	.423	.50 (.09-2.73)

Table 5 shows the results for Education risk practices with different predictor variables. The columns are showing the categories of predictor variables and rows are showing the outcomes variables categories followed with their OR, 95% CI for OR, and their P-values. The OR=1 presents the reference group OR. The yellow highlighted and (--) present that there was no significant association of those variables. The estimated OR of non-formal education is 2.29 times as large as in who do not hear IPM than in who heard IPM with 95%(1.46-3.36) of OR. They don't hear IPM showed high-risk levels being less likely to non-formal education (p-value=.000). The estimated OR of primary level education is 2.21 times as large as in who do not hear IPM than in who heard IPM with 95%(1.46-3.36) of OR. They do not hear IPM showed high-risk levels being less likely to primary level education (p-value=.000). Similarly estimated OR of middle-level education is 2.20 times as large as in who do not hear IPM than in who heard IPM with 95%(1.19-4.05) of OR. They do not hear IPM showed high-risk levels being less likely to middle-level education (p-value=.001). Similarly estimated OR of undergraduate level education is 2.66 times as large as in who do not hear IPM than in who heard IPM with 95%(1.24-5.73) of OR. They do not hear IPM showed high-risk levels being less likely to undergraduate level education (p-value=.012). Similarly estimated OR of graduate-level education is 2.77 times as large as in who do not hear IPM than in who heard IPM with 95%(1.29-5.95) of OR. They do not hear IPM showed much higher risk levels being less likely to graduate-level education (p-value=.009).

Label information on the containers of the insecticides for the preparation of required dose is a very crucial factor while most of the farmers were unable to read it as many are unable to

understand the terminologies and units $p<0.001$. There is a need to start a campaign to give awareness to farmers about it. Table 6

The regression analysis also showed that most common practice to control the pest was use of chemical abundantly by the farmers instead of alternative methods ($p= 0.001$).

Disposal of empty containers is a potential factor that can bring resistance in pests, pollution and outbreak of secondary pest. This practice an make the situation worse than sustainability ($p=0.001$). Use of protective equipment by the farmers is neglected most ($p=0.004$) Table 6.

**Crop Practices**	**Cultural Control**		1		1		1		1		1		1		1
	**Chemical Control**	.000	2.78 (1.72-4.48)	.090	1.73 (.918-3.27)	.39	1.44 (.61-3.37)	.166	2.00 (.75-5.32)	.006	3.228 (1.41-7.65)	.136	1.80 (.83-3.89)	.023	2.27 (1.11-4.61)
	**Biological Control**	.028	.43 (.20-.91)	.019	.26 (.08-.80)	.054	.22 (.04-1.02)	.	.	.118	.28 (.05-1.37)	.121	.40 (.12-1.27)	.	.
	**IPM**	.003	.26 (.10-.64)	.011	.20 (.05-.69)	.037	.11 (.01-.87)	.178	.33 (.06-1.65)	.069	.14 (.01-1.16)	.038	.20 (.04-.91)	.022	.09 (.01-.70)
**Disposal of empty containers**	**Government Collection**		1		1		1		1		1		1		1
	**Burry**	.000	63 (8.73-454-2)	.001	11 (2.58-46.7)	.054	4.5 (.97-20.8)	.080	4 (.84-18.8)	.005	18 (2.40-134.8)	.001	12 (2.83-50.7)	.002	23 (3.1-170.3)
	**Burnt**	.013	13 (1.70-99.3)	.423	2 (.36-10.9)	.423	2 (.36-10.9)	1	1 (.14-7.0)	.142	5 (.58-42.7)	.423	2 (.36-10.9)	.142	5 (.58-42.7)
	**Throw in Trash**	.001	26 (3.52-191.5)	.002	10 (2.3-42.7)	.038	5 (1.09-22.8)	.080	4 (.84-18.8)	.037	9 (1.14-71.03)	.423	2 (.36-10.91)	.050	8 (1-63.9)
**Training and Protective Measures**	**Hand Gloves**		1		1		1		1		1		1		1
	**Eye Glasses**	.000	.17 (.09-.34)	.001	.16 (.05-.46)	.013	.07 (.01-.58)	.017	.08 (.01-.64)	.058	.40 (.15-1.03)	.002	.10 (.02-.42)	.034	.38 (.16-.93)
	**Overall**	.000	.01 (.002-.12)	.	.	.013	.07 (.01-.58)	.017	.08 (.01-.64)	.009	.06 (.009-.50)	.003	.05 (.007-.37)	.	.
	**Respirator**	.000	.03 (.009-.14)	.002	.04 (.005-.29)	.	.	.	.	.007	.13 (.03-.58)	.	.	.	.
	**Face Mask**	.004	.52 (.33-.81)	.163	.64 (.34-1.2)	.533	.76 (.33-1.75)	.057	.33 (.10-1.03)	.226	.60 (.26-1.3)	.074	.50 (.23-1.06)	.136	.55 (.25-1.20)
	**Boot/Shoes**	.000	.05 (.01-.16)	.001	.08 (.01-.33)	.	.	.019	.16 (.03-.74)	.	.	.003	.05 (.007-.37)	.003	.11 (.02-.47)

Discussion

Current study reported that most of the farmers were male (93.6%) while 6.4 % were female. The study conducted by Dilek oztas et al in turkey reported 100% male farmers. Similar work done by Oluewe and c[illegible]e in Nigeria 93% of farmers were male. Lekei et al also reported the same trend 93% farmers were male.

The mean age of the farmers was in range of 31-50 years. The mean age of farmers was 18-51 years reported by Tuna et al in a study done about farmer’s knowledge, attitude and behavior about pesticides in Turkey. The average age of Farmers was 37.5 years according to lekei et al in Tanzania. Mubashar M et al conducted study on knowledge of farmers regarding pesticides usage and biosafety in Pakistan reported average age of farmers was 30-50 years. Similar trend was reported by Khuhru S N et al in northern Sindh Pakistan 18-50 years age of farmers.

Education level of farmers is closely related with their practices. Educated farmers can read and understands the instructions given on insecticides about dose preparation and precautionary measures given. The current survey showed 34% farmers were illiterate, 16% had primary level education, 8.33% had middle level education while 12.33 % were graduated from university and 11.33 % were those who get education up to higher and undergraduate level. A study conducted by Yassin et al on Knowledge, attitude, practices and toxic symptoms related to use of pesticides in philistine showed 8.5% were uneducated. In northren Sindh it was reported 12.2% were illiterate 40.9% were primary and 27.7% had middle level education. In lodhran and vehari Pakistan similar trend was reported 26.4% were illiterate. These results are in Accordance with Khan and Iqbal who reported that majority of farmers in Pakistan had low level of education only 6% had university level education.

Experience of farmers is very important in order to acquire skills. This factor alone can help to increase yield and make farming cost effective. Current study represented 35% have 7-9 years of farming experience, 13% have 10-12 years while 29% had 4-5years of farming experience. The same trend was reported by Mubasher M et al 41% had 11-15 years 37.9% having 5-10 years of farming experience. Research in Pakistan represented that sociodemographic characteristics of farmers like gender, age, education level and farming experience greatly affect the use of insecticides.

Alternative pest control method is very important component of IPM and very essential for sustainability. The result of current study shows 63% of respondents did not had any idea about alternative methods. Among 37% that know about alternative method crop rotation only practiced among farmers. Biological control is a very effective method along with the chemical control but unfortunately due to low level of knowledge and awareness 65.3% of respondents did not know about this. This unawareness leads to force them only rely on insecticidal control. Khan M et al reported few farmers implement IPM and most did not know about non chemical method. According to them cultural control and mechanical methods were easy to implement. Khusro N S et al reveals 22.72% farmers practiced cultural method, 22.72% could not identify pest and 95.45% did not use any other alternative method to control pest. The work is also supported by Yassin et al farmers did not have any knowledge about biological control or natural control agent.

Following the instruction given on the products for the preparation of proper dose is very important to prevent the overused as well as the release of residues in environment ultimately leads to development of resistance in insects. Present study reported that only 22% farmers were able to prepare the required dose while 58% farmers were unable to understand the terminologies

and language of the label instructions. There was strong association between farmer's knowledge and understanding the label information of insecticides products. It leads to the higher exposure via the increase emission rate. Dalamas and Khan reported that 73% farmers were unable to read label instruction on insecticides containers. Similar trend was reported by Mubasher M et al 48.2% were unable to read instructions on insecticides label. Shetty et al reported major factor behind this is illiteracy and lack of awareness.

Failure to use of protective measures was another problem linked with the hazardous impact of insecticides on environment. A study conducted in North Greece and India reported the significant association between knowledge and use of protective measures. Clark and collegues reported the poor trend about use of protective measures among the farmers in tropics due the hot and humid climatic conditions farmers feel discomfort during use of protective measures.

The use of protective measure become an important step for the sustainable use of insecticides likewise the IPM , irrigation method, use of less toxic agents and alternative pest control methods (use of biological agents) are the key component for sustainable agriculture. In this survey it was found that most of the farmers neglected the use of protective gears.29.7% use face mask and 3.3% use long shoes. The use of respirator was rear during spray only 1.7% reported.40% farmers in Iran were reported to use any protective equipment during spray reported by Hashemi et al .

Furthermore improper disposal of empty containers may be another source of pesticides exposure. Mostly farmers dumped the empty insecticides containers in unsafe manners this leads to the contamination of environment due to run off, leaching and arial distribution of residues even in developing countries the practices were very common. Study in Tanzania and south Africa reported that farmers made use of empty insecticides containers for domestic purpose.

Data reveals most of respondents had no trainings from extension officer about the use of insecticides as Aldose *et al* (2018) 79.5% of farmers did not received any training whereas 12.3%, 5.1% and 3.1% received 2 to 4 and more than four trainings respectively. Similarly according to Aslam *et al* (2007) farmers of Pakistan had low level of knowledge about insecticides use however training by extension officer can be an effective step to reduce risk factors[7]. According to another study conducted in Ethiopia by Negatu *et al* (2016) 85% of the farmers had no any training regarding application of insecticides [8], while Ibitayo (2006) reported similar trend of farmers [4]. According to this study 98% of farmers did not had any training about insecticides application. In UK a study conducted regarding risk assessment about safe use of insecticides among workers. It indicated that proper training and adoption of proper safety measures can reduce the health hazards [9].

Conclusion

The findings of this survey demonstrate poor understanding and risky practices adopted by the farmers are the major contributing factors towards resistance development and health issues. For underdeveloped countries like Pakistan a comprehensive and well planned program targeting on alternative pest control method and use of biological agents needed to be initiated that can reduce the total dependency on chemicals.

There is a dire need to start up the farmer’s awareness campaigns as well as need to organize farmers by giving them trainings about proper disposal of empty containers to minimize the resistance development and resurgence of secondary pests.

Acknowledgement

The author sincerely thanks to the farmers and extension agriculture department for participating and contributing to this study. Special thanks to Dr Fayyaz Ahmed (Statistics department), University of Gujrat for his kind help in statistical analysis.

Conflict of interest

There is no conflict of interest.

References

1. Rodrigues S R A, spindola F A, Torres B J, Herbert A A S, colares F. response of different populations of seven lady beetle species to Lamda-Cyhalothrin with record of resistance. Ecotoxicology and environmental safety 2013; 96 , 53-60.
2. Abbas N, Shad S A , Ismail M, resistance to conventional and new insecticides in houseflies (diptera: muscidae) from poultry facilities in Punjab pakistan. J. Econ. Entomol.2014; 108: 826-833
3. Basit M, Saeed S, saleem MA ,Denholm I, Shah M. Detection of resistance , cross resistance and stability of resistance to new chemistry insecticides in helicoverpa armigera. J Econ Entomol 2013; 106:1414-1422.
4. Ibitayo OO, Egyptian farmers,attitude and behavior regarding aricuktural pesticides: implications for pesticides risk communication. Risk anal 2006; 26(4) 989-995.
5. Sodavy p, sitha M, Nugent r, Murphy h. situation analysis: farmers awareness and perceptions of the effects of pesticides on their health .field document.combodia :FAO Community IPM PROGRAM .2000

6. Sankoh AI, Whittle R, Semple KT, Jones KC, Sweetman AJ(2016) An assessment of the impacts of pesticide use on the environment and health of rice farmers in Sierra Leone.Environment International 94: 458-466.
7. Aslam M, Ali T, Zafar MI, Ahmad M (2007) Training needs offruit growers regarding pesticide use for sustainable environmental health in Punjab. Pakistan Journal of Agricultural Sciences 44: 511-518.
8. Negatu B, Kromhout H, Mekonnen Y, Vermeulen R (2016) Use of Chemical Pesticides in Ethiopia: A Cross-SectionalComparative Study on Knowledge Attitude and Practice of Farmers and Farm Workers in Three Farming Systems. AnnualOccupation Hygiene 16
9. Remoundou K, Brennan M, Hart A, Frewer LJ (2014) PesticideRisk Perceptions, Knowledge, and Attitudes of Operators,Workers, and Residents: A Review of the Literature. Human and Ecological Risk Assessment 20:1113 –1138
10. Khuhro, S.N., I.A. Junejo, M.H. Hullio, M.F. Hassan, S.A. Maitlo and M.A. Shaikh. 2020. Knowledge attitude practice regarding pesticide application among vegetable growers of Dadu canal irrigated areas of northern Sindh Pakistan. *Pakistan Journal of Agricultural Research*, 33(2): 331-336.
11. Khan, M., H.Z. Mahmood and C. Damalas. 2015. Pesticide use and risk perceptions among farmers in the cotton belt of Punjab, Pakistan. Crop Prot. 67: 184-190. https://doi.org/10.1016/j.cropro.2014.10.013.
12. Yassin, M.M., M.T.A. Abu and J.M. Safi. 2002. Knowledge, attitude, practice, and toxicity symptoms associated with pesticide use among farm workers in the Gaza Strip. Occup. Environ. Med., 59: 387–393. https://doi.org/10.1136/oem.59.6.387.

13. PH. Shetty, M. Murugan, MB. Hiremath, and KG. Sreeja, “Farmerss education and perception on pesticide use and crop economies,” in *in Indian agriculture. JES*, vol. 1, p. 3, 3-8, 1(1, 2010.

14. S. H. Hashemi, S. M. Hosseini, and M. K. Hashemi, “Farmer’s perceptions of safe use of pesticides: Determinants and training needs,” *International Archives of Occupational and Environmental Health*, vol. 85, pp. 57–66, 2012.

15. Mubasher M, aldosari F O, Baig M B, Altotaibi M, Khan A Q, “Assessment of farmers on their knowledge regarding pesticides usageand biosafety”, 2019.

16. Dalamas AC, Khan M, “ Farmers knowledgeabout common pest and pesticides safety in conventional cotton production in Pakistan 2015, (77), 45-51.

17. Oztas D, Kurt B, Koc aysegul, Akbaba M and Ilter H, Knowledge level, attitude and behavior of farmers in Cukurora region regarding the use of pesticides.” Biomed research international, Vol 2018.

18. Oluwole O, Cheke R A, Health and environmental impact of pesticide use practices: A case study of farmers in Ekiti state Nigeria.” International journal of agricultural sustainability, 2009. vol. 7,(3),153-163.

19. Tuna RY, Gun I, Ceyhen O, “knowledge,Attitude and Behaviour of farmers on storage conditions and safety use of insecticides.

20. Lekei E E, Ngowi A V, London L.” Farmers knowledge, practices and injuries associated with pesticides exposure in rural farming villages in Tanzania,” BMC public Health, 2014, vol. 14, 1-13.

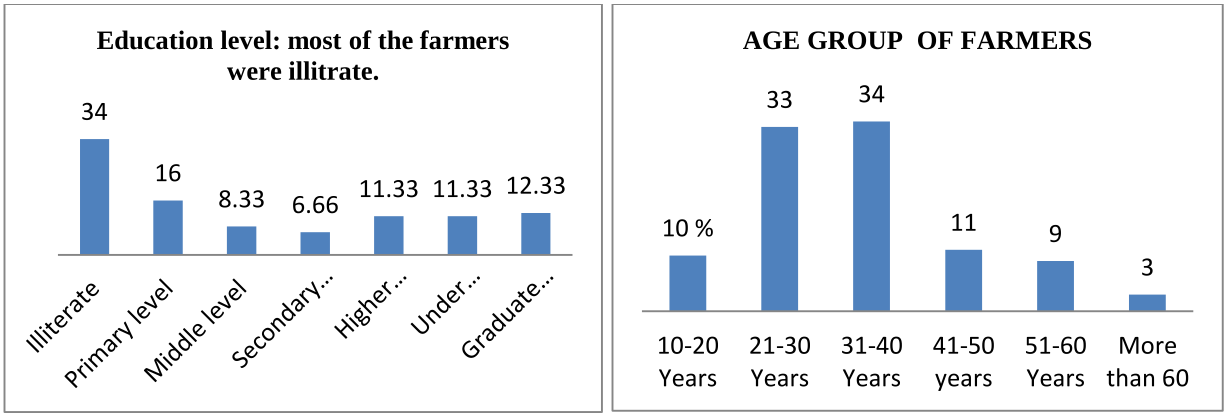

Figure 01 a: Education level of the farmers represented in fig 01 (a).percentage of farmers was given on Y-axis while their education level was placed on X-axis.Fig 01(b): Age group of farmers was represented on x-axis and their percentage was given on Y-axis.

Click here to access/download
Supporting Information
bakht paper submitted to plos one highlited.docx

A Cross Sectional Survey of Knowledge, Attitude and Practices Related to the Use of Insecticides among Farmers in Industrial Triangle of Punjab, Pakistan

Bakhtawer, Sumera Afsheen

Department of Zoology, University of Gujrat, Hafiz Hayat Campus, Jalalpur Jattan Road, Gujrat, Punjab 50700, Pakistan.

E-mail: *sumera.afsheen@uog.edu.pk*

Abstract

Pesticides in Pakistan are abundantly utilized for pest control in agriculture sector. The over and unsafe of insecticides plus poor handling, leads to the development of resistance, outbreak of secondary pests and hazardous impact on environment. The present study was aimed to access the current knowledge, attitude and common practices of farmers about the use of insecticides against pest in industrial triangle of Province Punjab, Pakistan. This study was conducted during October and December, 2019. In this study farmers (n=300) took part from three localities of Province, Punjab (Gujrat, Gujranwala and Sialkot). Farmers were interviewed using a questionnaire to collect data about the knowledge of pest control by use of chemical method, biological method and combination of both as well as utilization of predators to eradicate the pests. The result shows almost all (93%) farmers were male and they did not know about the insecticides mode of action and its chemical composition. They also do not have any knowledge about the biological control of pests. Farmers did not get any assistance or help from

Agriculture Extension Officer. They even did not properly dispose off the empty containers of insecticides. Statistical analysis reveals that lack of education and awareness about biological control of pest depicts development of resistance and outbreak of secondary pest including health hazards and environmental pollution. Poor understanding about pests, abundant use of insecticides, incorrect perception about application of insecticides and negligence regarding biological control shows there is need to initiate public awareness programs to ensure the application of IPM and sustainable agriculture.

Formatted: Indent: First line: 0"

The present study **was** aimed to access the current knowledge, attitude and common practices of farmers about the use of insecticides against pest in industrial triangle of **Province** Punjab**,** Pakistan. **This study was conducted during October and December, 2019**. **In this study farmers (n=300) took part from three localities of Province, Punjab** (Gujrat, Gujranwala and Sialkot). **Farmers were interviewed using a questionnaire to collect data about the knowledge of pest control by use of chemical method, biological method and combination of both as well as utilization of predators to eradicate the pests**. ANOVA was used to investigate potential predicators. The result shows almost all (93%) farmers were male and they did not know about the insecticides mode of action and its chemical composition. They also do not have any knowledge about the biological control of pests. Farmers did not get any assistance or help from **Agriculture Extension Officer**. They even did not properly **dispose off** the empty containers of insecticides. Statistical analysis reveals that lack of education and awareness about biological control of pest depicts development of resistance and outbreak of secondary pest including health hazards and environmental pollution.

Poor understanding about pests, abundant use of insecticides, incorrect perception about application of insecticides and negligence regarding biological control shows there is need to initiate public awareness programs to ensure the application of IPM and sustainable agriculture.

Introduction

Being an agricultural country economy of Pakistan based on agriculture sector.1.8 billion people round the globe deal with pesticides to increase the yield of crops and protect them from pest attack. 42.5 percent people in rural areas of Pakistan involved in agricultural activities. Chemical control of pests in agriculture sector is the major prominent method to maximize productivity of crops.

Various chemicals including Organophosphate, Neonicotinoids, Carbamates and Pyrethoids are abundantly used. 108 types of insecticides, 39 types of herbicides,6 types of rodenticides and 5 types of aracicides were reported to be used in Pakistan for different crops. E use of pesticides have increased 9% or more per hacter in most developing countries including Pakistan.

It has been observed that farmers mostly rely on chemicals to protect their crops from pest's attacks without knowing the harmful consequences of insecticides. This practice leads not only to development of resistance in pests but also reduce the number of useful insects, outbreak of secondary pests, health issues and environmental pollution [2].

In Province Punjab variety of crops are cultivated including wheat, Rice, Cotton, Sugarcane, fruits and vegetables. In these crops field sap sucking insects cause damage to crops at all stages of crop development. Farmers of this region rely on insecticidal control of pests [3]. These farmers did not have any awareness about the chemical nature of insecticides [4]. They also do

not have any assistance or training from agricultural extension sector regarding application of insecticides [5]. Due to lack of knowledge about harmful results of pesticides farmers did not pay attention about taking precautionary measures. Improper disposal of Empty containers that contain surplus enough quantity of pesticides leads to the pollution as well as resistance development in non target species.

For sustainable agriculture there is need to reduce the use of insecticides and control the pest attack by other means. It is very necessary to adopt the biological control method along with chemical control in order to bring sustainability. For this purpose public awareness is very important to give general knowledge to farmers about the use of pesticides. Integration of chemical control with biological control is the basic part of IPM but unfortunately it has been rarely applied [1]. Use of natural predators can be economical to farmers as well as it is ecofriendly.

By keeping in view all the factors current study was designed to access the knowledge of farmers about pesticides application and alternative methods to control pests. This will help the agriculture department to design a comprehensive plan to educate farmers as well as built up an effective IPM strategy to control pests attack, secure food and bring sustainability.

Chemical control of pests in agriculture sector is the major prominent method to get higher yield of crops. Various chemicals including Organophosphate, **Neonicotinoids**, **C**arbamates and **P**yrethoids are abundantly used. Integration of chemical control with biological control is the basic part of IPM but unfortunately it has been rarely applied [1].

It has been observed that farmers mostly rely on chemicals to protect their crops from pest's attacks without knowing the harmful consequences of insecticides. This practice leads not only **to** development of resistance in pests but also reduce the number of useful insects, outbreak of secondary pests, health issues and environmental pollution [2].

In **Province** Punjab variety of crops are cultivated including wheat, Rice, Cotton, Sugarcane, fruits and vegetables. In these crops field sap sucking insects cause damage to crops at all stages of crop development. Farmers of this region rely on insecticidal control of pests [3]. These farmers did not have any awareness about the chemical nature of insecticides [4]. They also do not have any assistance or training from agricultural extension sector regarding application of insecticides [5]. For sustainable agriculture there is need to reduce the use of insecticides and control the pest attack. It is very necessary to adopt the biological control method along with chemical control in order to bring sustainability. For this purpose public awareness is very important to give general knowledge to farmers. Now they can use predators to control the pest. Use of natural predators can **be** economical to farmers as well as it is ecofriendly.

Material and Method

A cross sectional study was conducted during October 2019 to February 2020 by using questionnaire based interview of farmers. In this survey face to face conversation was done with farmers. Firstly in local language aim of study was described later on after their willingness questions were asked verbally and their response was noted on questionnaire. This survey was carried out in industrial triangle including Gujrat, Gujranwala and Sialkot. These sites were selected on the basis of operational convenience and industrial importance of this region. Due to availability of water and suitability of climatic conditions various crops like wheat, rice, fruits

and vegetables cultivated in this region. Farmers of this region use insecticides abundantly in this region to protect their crops from pests. The demographic characteristics of this region are given in Table 1.

**Province**	**District**	**Area Sq km**	**Population**		
			**Number**	**Rural %**	**Urban %**
Punjab	Gujranwala	3622	5014196	41.18	58.82
	Gujrat	3192	2756110	96.98	03.02
	Sialkot	3016	3893672	70.64	29.36

Table 1: Demographic characteristic of study area source: Pakistan bureau of statistics 2017.

From Each site six localities were further selected randomly. From Gujrat six localities include Jalal pur jattan (JPJ), Karianwala (KN), Dolat Nagar (DN), Wazirabad (w), Lalamusa (LM) and Kharian (KH). From Gujranwala site ferozawala (FW), Uggochak (UG), Veropal Chatha (VC), Ali pur Chatha (AC), Qila miyan singh (QS) and Kot Ladha (KL) were selected for data collection. Daska (D), sambrial (S1), Sahowal (S2), Majra Kalan (MK), Dera sanda (SD) and Sahowala (S3) from Sialkot site were included in survey study. Detail of study area presented in Figure 01.

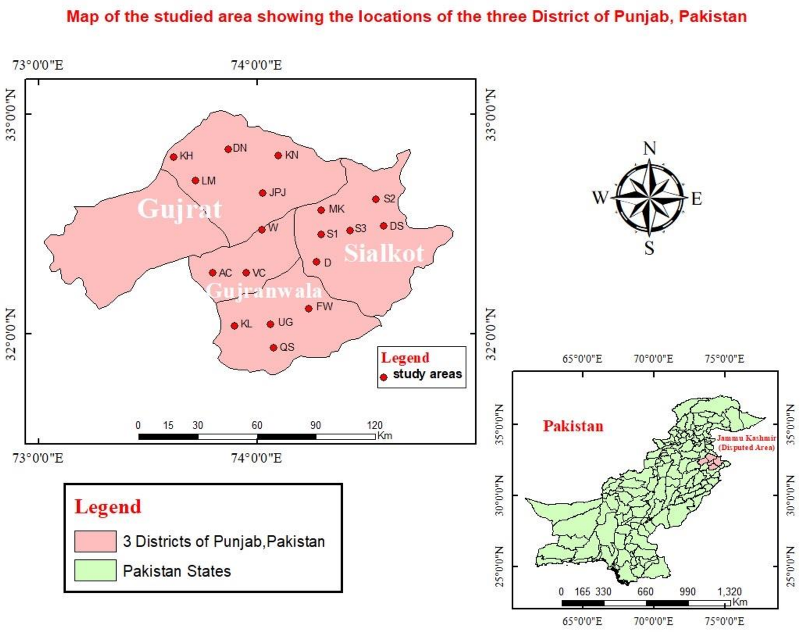

Map indicating current study area (Map created by using GIS-ARc).

In first step questionnaire was prepared and survey was conducted from a sub sample of 20 Farmers who were reluctant to participate in study. On the basis of results of pretest this survey was further modified to reduce the deficiencies felt at that time. In second step a comprehensive survey was conducted among 300 farmers of District Gujrat, Gujranwala and Sialkot of Punjab province.

A structured questionnaire having open ended and close ended questions was used to collect information by using Urdu and Punjabi Language. The questionnaire consist up of three sections, First part is related to sociodemographic characters of farmers such as Gender, marital status,

Age, Education level , Farm area, irrigation method, farming Experience and working hours they spend in their crops were determined. Second part related to perceptions like which insecticide is better and they prefer to use more, which crop and which pest most frequently targeted, how they prepare the spray dose for targeted pests, they have any idea about alternate pest control method like IPM, Biological agent or natural enemies and third part related to the attitude and practices of farmers like how hey protect themselves during spray, they use protective equipment or not and they acquired any skill or training from any.

This study was conducted industrial triangle of Punjab (Gujrat, Gujranwala and Sialkot) Pakistan.

Study area

A cross sectional study was conducted during OCT-DEC 2019 by using questionnaire based interview of farmers. This survey was carried out in industrial triangle including district Gujrat, Gujranwala and Sialkot Fig 01. These districts were selected on the basis of **operational** convenience and industrial importance of this region. The demographic characteristics of this region are given in Table 1.

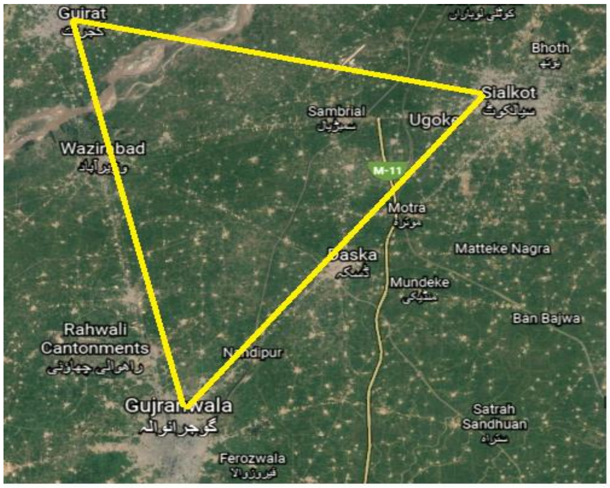

Map indicating current study area (Map created by using Google Maps).

Province	District	Area<br>Sq km	Population		
			Number	Rural %	Urban %
Punjab	Gujranwala	3622	5014196	41.18	58.81
	Gujrat	3192	2756110	96.98	30.02
	Sialkot	3016	3893672	70.64	29.36

Table 1: Demographic characteristic of study area source: Pakistan bureau of statistics 2017.

In first step questionnaire was prepared and survey was conducted from a sub sample of 20 Farmers who were reluctant to participate in study**. On the basis of results of pretest this survey was further modified to reduce the deficiencies felt at that time**. In second step a comprehensive survey was conducted among 300 farmers of **D**istrict Gujrat, Gujranwala and Sialkot of Punjab province.

A structured questionnaire having open ended and close ended questions was used to collect information by using Urdu and Punjabi Language. The questionnaire consist up of three sections, First part is related to sociodemographic characters of farmers. Second part related to perceptions and third related to the attitude and practices of farmers.

Interview procedure

Each village was visited for 4-5 days and 10-15 farmers were interviewed per day. Before interview main objectives of research were explained to them and later on voluntary they involved giving information.

Ethics Statement

Before interview main objectives were explained clearly to farmers. Later on by their willingness data was recorded on questionnaire by verbally asking the questions because majority of farmers were not able to read and understand questionnaire. This study was approved by all statuary forums of the University of Gujrat, Gujrat, Pakistan including the Departmental Review and Research Committee (DRRC), Advanced Study and Research Board (ASRB), Board of Faculty (BoF) and Academic Council.

Results

A total of 300 farmers from three Districts were participated in the study.

Results

A total of 300 farmers from three Districts were participated in the study. Random sampling was done. Most of the farmers were involved in cultivation of wheat, rice, millet, sorghum and vegetables in this region. The response rate was 100% farmers did not refuse to participate.

Formatted: Indent: First line: 0.5"

Formatted: Font: 12 pt, Not Bold

Demographic characteristics of Respondents

The majority of farmers were male (93.66 %) and 34% had no formal education. 11.33% **of** respondents had education level up to higher secondary school and only 12.33% Graduated. Most of the farmers were in 31-40 (34 %) year's age. Detail of demographic characteristics of farmers is given in Table 2.

Table 2: Sociodemographic characteristics of farmers in three localities of province Punjab Pakistan (n= 300)

Gender	Character	Number	% age
	Male	281	93.66
	Female	**19**	6.33
Age	10-20	30	10
	21-30	99	33
	31-40	102	34
	41-50	33	11
	51-60	27	9
	More than 60	9	3
Marital status	Single	33	11
	Married	255	85
	Divorced/ widowed	12	4
Education level	Illiterate	102	34

	Primary level	48	16
	Middle level	25	8.33
	Secondary level	20	6.66
	Higher secondary level	34	11.33
	Under graduate level	34	11.33
	Graduate level	37	12.33

Demographic characteristics of Respondents

The majority of farmers were male (93.66 %) while only 5.4% women were involved in farming. Education is a very important variable for the assessment of farmer's knowledge and practices. It was founded that 34% farmers were illiterate, 16% had primary level, 6% had middle level education, 11.33% of respondents had education level up to higher secondary school and only 12.33% were graduated. Figure 02 represents the detail of the education level of farmers. Age group of the farmers ranged from 20-60 years with an average of 31-40 (34 %) years. Detail of age in years was represented in figure 02.

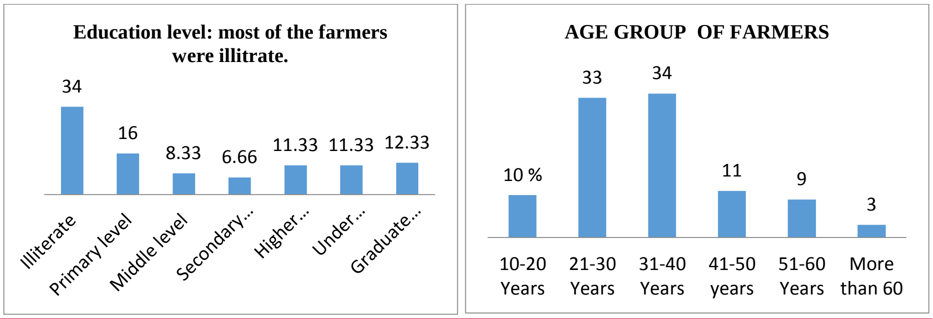

Figure 02: Education level and age group of farmers

Knowledge of respondents

Most of the farmers have 1 hector area for the cultivation of crops. This data indicate that these farmers had small farming units. Most common irrigation method adopted by the farmers was tube well. 7% farmer used sprinkle method while only 3% utilized canal system in the region for irrigation.

38 % farmers spend 4-6 hours for farming activities daily. Only 2% reported to do work more than 12 hours. Most of the respondents had 7-9 years of farming experience. (Table 3)

Table 3: knowledge of farmers in three localities of province Punjab Pakistan (n= 300)

**Characters**		**Number**	**% age**
Farm size (ha)	Less than 1	87	29
	1-1/2	123	41
	2	48	16
	More than 2	42	14
Irrigation method	Drip method	54	18
	Sprinkle	21	7
	Tube well	216	72
	Canal system	9	3
Farming experience	1-3 years	54	18
	4-6 years	87	29
	7-9 years	105	35
	10-12 years	39	13
	More than 12	15	5
Working hours	1-3 hours	87	29
	4-6 hours	114	38
	7-9 hours	57	19
	10-12 hours	36	12
	More than 12 hours	6	2

Knowledge of respondents regarding pesticides

Most of the insecticides used by the farmers were belong to Acetamprid, Cypermethrim, Lambda Cyhalothrin and Dimethoate group. Detail information about insecticide is given in Table 4.

Table 4: Knowledge of farmers in three localities of province Punjab Pakistan (n= 300)

Knowledge of farmers			
Pesticides used	Acetamprid<br>Cypermethrin<br>Lamda cyhalothrin<br>Dimethoate		
Characters		Number	% age
Crops	Wheat	93	31
	Rice	69	23
	Sugarcane	3	1
	Millet	6	2
	Sorghum	12	4
	Vegetables	117	39
Pests	Sumuts	30	10
	Aphids	147	49
	Whiteflies	36	12
	Jassids	42	14
	Mildew	30	10
	Mealybugs	9	3
	Termites	6	2
Frequency of spray	Two time	44	14.66
	Three time	139	46.33

	Four time<br>Five times	97<br>20	32.33<br>6.66
Stage of crop	Early stage<br>Grown stage<br>Mature stage<br>Harvesting stage	202<br>56<br>25<br>17	67.33<br>18.66<br>8.33<br>5.66
Mode of application	Arial<br>Mix with water<br>Synergist	214<br>40<br>46	71.33<br>13.33<br>15.33
Lebal instructions	Follow<br>Proper dose<br>Schedule<br>Maintain containers	126<br>66<br>45<br>63	42<br>22<br>15<br>21
Alternative synthetic insecticides	Yes<br>No	111<br>189	37<br>63
In case yes	Crop rotation<br>Organic farming<br>Crop rotation<br>Crop mixtures	3.33<br>1.11<br>96.57<br>7.77	3<br>1<br>87<br>7
Knowledge about IPM	Yes<br>No	94<br>206	31.33<br>68.66
Biological control	Yes<br>No	104<br>196	34.66<br>65.33
Knowledge about natural enemies	Yes<br>No	99<br>201	33<br>67

**Table 3: Knowledge of farmers in three localities of province Punjab Pakistan (n= 300)**	
Knowledge of farmers	
Insecticides used	Acetamprid

		Cypermethrin<br>Lamda cyhalothrin<br>Dimethoate		
Characters			Number	Percentage
Crops		Wheat<br>Rice<br>Sugarcane<br>Millet<br>Sorghum<br>Vegetables	93<br>69<br>3<br>6<br>12<br>117	31<br>23<br>1<br>2<br>4<br>39
Pests	Scientific name	Common name		
	Ustilaginaceae<br>*Aphidoidea*<br>*Aleyrodidae*<br>*Amrasca biguttula*<br>*Golovinomyces orontii*<br>*Pseudococcidae*<br>*Isoptera*	Sumuts<br>Aphids<br>Whiteflies<br>Jassids<br>Mildew<br>Mealybugs<br>Termites	30<br>147<br>36<br>42<br>30<br>9<br>6	10<br>49<br>12<br>14<br>10<br>3<br>2
Frequency of spray (per Month)		Two time<br>Three time<br>Four time<br>Five times	44<br>139<br>97<br>20	14.66<br>46.33<br>32.33<br>6.66
Stage of crop		Early stage<br>Grown stage<br>Mature stage<br>Harvesting stage	202<br>56<br>25<br>17	67.33<br>18.67<br>8.33<br>5.67
Mode of application		Arial<br>Mix with water<br>Synergist	214<br>40<br>46	71.33<br>13.33<br>15.33
Lebal instructions		Follow<br>Proper dose<br>Schedule<br>Maintain containers	126<br>66<br>45<br>63	42<br>22<br>15<br>21
Alternative synthetic insecticides		Yes<br>No	111<br>189	37<br>63
In case yes		Crop rotation<br>Organic farming<br>Crop rotation<br>Crop mixtures	3.33<br>1.11<br>96.57<br>7.77	3<br>1<br>87<br>7
Knowledge about IPM		Yes<br>No	94<br>206	31.33<br>68.67
Biological control		Yes	104	34.67

	No	196	65.33
Knowledge about natural enemies	Yes<br>No	99<br>201	33<br>67

The insecticides were used during the cultivation of different crops include wheat, rice, sugarcane, millet, Sorghum and vegetables. Highest proportion of these insecticides 39% were used for vegetables followed by wheat 31% and Rice 23% .Major pest reported for these crops were smuts, Aphids, whiteflies, Jessed, mildew, mealy bugs and termites. Out of which 49% of insecticides were used to control aphids. These insecticides were sprayed three (46.33%) to four (32.33%) times by majority of farmers. These sprays were used during the early stage of pest growth (67.33%). Only 5.66% were reported to use spray at harvesting stage of pests. 71.33% of farmer sprays their crops by Arial method, 15.33% used synergist and only 13.33% used mixture with water. Label instruction for preparing dose is very crucial step to properly use insecticides against pests. 42% of respondents know to **understand** the label instruction during preparation for spray. 22% of respondents were able to made proper dose. 15% of the respondents follow the schedule for spray and 21% were founded to maintain empty containers of insecticides.

When the farmers were asked about the alternative synthetic insecticides, 63 % responded No. 68.6% did not have knowledge about IPM. Most of the respondent 65.3% did not know about biological control method of pests.

Common practices of farmers

Control of pest by the use of chemicals 55.33% was common practice followed by the cultural control methods (32.33%). Only a small fraction of respondents have adopted IPM 5.33% and biological control method 7 % in their farming routines. These farmers 50.33 % burry

the empty containers and 14% of respondents burnt them while 31% throw them in trash without any processing. As well as precautionary measures are concerned, hand gloves 44% and facemask 41% or covering the face with cloth are most adopted precautionary measures. use of glasses, overall, respirator and long shoes were mostly neglected measures among farmers during the use of insecticides. For the proper use of insecticides, training is very important but unfortunately 7% of respondents have any acquired skill and 12% have training about the use of insecticides while most of them get name of products and use them for pests just by asking from agriculture officer.

Table 5: Common practices of farmers in three localities of province Punjab Pakistan (n= 300)

	Character	Number	%age
Crop practices methods	Cultural	97	32.33
	Chemical	166	55.33
	Biological	21	7
	IPM	16	5.33
Practices for Disposal of insecticides containers	Govt collection	11	3.66
	Burry	151	50.33
	Burnt	43	14.33
	Throw in trash	95	31.66
Farmer training /skill	Pesticides application	36	12
	Agriculture officer guidance	243	80
	Use acquired skills	21	7
Practice to use Protective measures	Hand gloves	132	44
	Glasses	33	11
	Overall	3	1
	Respirator	3	1

	Face mask<br>Boot/shoes	123<br>6	41<br>2
Time of spray	Morning<br>Noon<br>After noon<br>Evening	132<br>6<br>111<br>21	44<br>2<br>37<br>7

Univarient regression model showed farmers having non formal education **are at** high level of risk as compared to the respondents that know about the mode of action of insecticides. There was strong correlation between education level and common practices of farmers. However knowledge about the IPM and biological control method of pests was very low indicated the need of awareness campaign among farmers. Chemical control was the dominated method practiced among the farmers for the control of pests.

The univariate regression analysis showed that all factors had significant differences with the education level of farmers (p< 0.001). Table shows the knowledge of farmers about the alternative methods like IPM, Biological control and natural enemies is very poor however the farmers having knowledge about them are more likely to reduce the harmful impact of insecticides.

Table 05: Summary of univariate regression analysis column of the table refers to the education level of the farmers and rows refer the practices of farmers. Shown are the odd Ration (OR) and 95% confidence interval (95% CI) .

		P-value	OR (95%CI)	P-value	OR (95%CI)	P-value	OR (95%CI)	P-value	OR (95%CI)	P-value	OR (95%CI)	P-value	OR (95%CI)	P-value	OR (95%CI)
**Heard IPM**	**Yes**		1		1		1		1		1		1		1
	**No**	.000	2.29 (1.46-3.36)	.000	2.21 (1.46-3.36)	.011	2.20 (1.19-4.05)	.079	2.12 (.91-4.92)	.655	1.22 (.50-2.94)	.012	2.66 (1.24-5.73)	.009	2.77 (1.29-5.95)
**Biological Control**	**Yes**				1		1		1		1		1		1
	**No**	.000	2.43 (1.59-3.72)	.000	2.433 (1.59-3.72)	.011	2.200 (1.19-4.05)	.034	2.57 (1.07-6.15)	.187	1.85 (.74-4.65)	.061	2.00 (.97-4.12)	.004	3.25 (1.47-7.17)
**Natural Enemies**	**Yes**				1		1		1		1		1		1
	**No**	.025	1.57 (1.06-2.34)	.025	1.57 (1.06-2.34)	.011	2.20 (1.19-4.05)	.002	5.25 (1.80-15.29)	.374	1.50 (.61-3.67)	.002	3.71 (1.61-8.55)	.494	1.26 (.64-2.49)
**Follow label Information B8[a]**	**Follow**		1		1		1		1		1		1		1
	**Proper Dose**	.03	.59 (.36-.96)	.08	.52 (.25-1.08)	.06	.30 (.08-1.09)	.01	.15 (.03-.68)	.32	.66 (.30-1.48)	.12	.52 (.23-1.18)	.28	.35 (.13-.89)
	**Follow Schedule of spray**	.001	.40 (.23-.78)	.004	.23 (.09-.63)	.206	.50 (.17-1.46)	.014	.15 (.03-.68)	.011	.20 (.05-.69)	.016	.29 (.10-.79)	.028	.35 (.13-.89)
	**Maintenance of Pesticide Container**	.000	.34 (.19-.61)	.08	.52 (.25-1.08)	.46	.70 (.26-1.08)	.02	.23 (.06-.81)	.01	.26 (.08-.80)	.006	.17 (.05-.60)	.079	.47 (.20-1.09)
**Irrigation method**	**Drip**		1		1		1		1		1		1		1
	**Sprinkler**	.40	.42 (.18-.96)	.05	.22 (.04-1.02)	.	.	.	.	.73	1.25 (.33-4.65)	.142	.20 (.02-1.71)	.739	1.25 (.33-4.65)
	**Tube Well**	.000	3.78 (2.28-6.28)	.000	3.88 (1.86-8.09)	.006	4.0 (1.50-10.65)	.18	1.85 (.74-4.65)	.001	6.0 (2.08-17.29)	.001	5.20 (1.99-13.54)	.000	6.5 (2.26-18.62)
	**Canal Irrigation**	.005	.21 (.07-.61)	.054	.22 (.04-1.02)	.	.	.	.	.	.	.273	.40 (.07-2.06)	.423	.50 (.09-2.73)

Table 5 shows the results for Education risk practices with different predictor variables. The columns are showing the categories of predictor variables and rows are showing the outcomes variables categories followed with their OR, 95% CI for OR, and their P-values. The OR=1 presents the reference group OR. The yellow highlighted and (--) present that there was no significant association of those variables. The estimated OR of non-formal education is 2.29 times as large as in who do not hear IPM than in who heard IPM with 95%(1.46-3.36) of OR. They don't hear IPM showed high-risk levels being less likely to non-formal education (p-value=.000). The estimated OR of primary level education is 2.21 times as large as in who do not hear IPM than in who heard IPM with 95%(1.46-3.36) of OR. They do not hear IPM showed high-risk levels being less likely to primary level education (p-value=.000). Similarly estimated OR of middle-level education is 2.20 times as large as in who do not hear IPM than in who heard IPM with 95%(1.19-4.05) of OR. They do not hear IPM showed high-risk levels being less likely to middle-level education (p-value=.001). Similarly estimated OR of undergraduate level education is 2.66 times as large as in who do not hear IPM than in who heard IPM with 95%(1.24-5.73) of OR. They do not hear IPM showed high-risk levels being less likely to undergraduate level education (p-value=.012). Similarly estimated OR of graduate-level education is 2.77 times as large as in who do not hear IPM than in who heard IPM with 95%(1.29-5.95) of OR. They do not hear IPM showed much higher risk levels being less likely to graduate-level education (p-value=.009).

Label information on the containers of the insecticides for the prepration of required dose is a very crucial factor while most of the farmers were unable to read it as many are unable to

understand the terminologies and units $p<0.001$. There is a need to start a campaign to give awareness to farmers about it. Table 6

The regression analysis also showed that most common practice to control the pest was use of chemical abundantly by the farmers instead of alternative methods ($p= 0.001$).

Disposal of empty containers is a potential factor that can bring resistance in pests, pollution and outbreak of secondary pest. This practice an make the situation worse than sustainability ($p=0.001$). Use of protective equipment by the farmers is neglected most ($p=0.004$). table 6.

**Crop Practices**	**Cultural Control**		1		1		1		1		1		1		1
	**Chemical Control**	.000	2.78 (1.72-4.48)	.090	1.73 (.918-3.27)	.39	1.44 (.61-3.37)	.166	2.00 (.75-5.32)	.006	3.228 (1.41-7.65)	.136	1.80 (.83-3.89)	.023	2.27 (1.11-4.61)
	**Biological Control**	.028	.43 (.20-.91)	.019	.26 (.08-.80)	.054	.22 (.04-1.02)	.	.	.118	.28 (.05-1.37)	.121	.40 (.12-1.27)	.	.
	**IPM**	.003	.26 (.10-.64)	.011	.20 (.05-.69)	.037	.11 (.01-.87)	.178	.33 (.06-1.65)	.069	.14 (.01-1.16)	.038	.20 (.04-.91)	.022	.09 (.01-.70)
**Disposal of empty containers**	**Government Collection**		1		1		1		1		1		1		1
	**Burry**	.000	63 (8.73-454-2)	.001	11 (2.58-46.7)	.054	4.5 (.97-20.8)	.080	4 (.84-18.8)	.005	18 (2.40-134.8)	.001	12 (2.83-50.7)	.002	23 (3.1-170.3)
	**Burnt**	.013	13 (1.70-99.3)	.423	2 (.36-10.9)	.423	2 (.36-10.9)	1	1 (.14-7.0)	.142	5 (.58-42.7)	.423	2 (.36-10.9)	.142	5 (.58-42.7)
	**Throw in Trash**	.001	26 (3.52-191.5)	.002	10 (2.3-42.7)	.038	5 (1.09-22.8)	.080	4 (.84-18.8)	.037	9 (1.14-71.03)	.423	2 (.36-10.91)	.050	8 (1-63.9)
**Training and Protective Measures**	**Hand Gloves**		1		1		1		1		1		1		1
	**Eye Glasses**	.000	.17 (.09-.34)	.001	.16 (.05-.46)	.013	.07 (.01-.58)	.017	.08 (.01-.64)	.058	.40 (.15-1.03)	.002	.10 (.02-.42)	.034	.38 (.16-.93)
	**Overall**	.000	.01 (.002-.12)	.	.	.013	.07 (.01-.58)	.017	.08 (.01-.64)	.009	.06 (.009-.50)	.003	.05 (.007-.37)	.	.
	**Respirator**	.000	.03 (.009-.14)	.002	.04 (.005-.29)	.	.	.	.	.007	.13 (.03-.58)	.	.	.	.
	**Face Mask**	.004	.52 (.33-.81)	.163	.64 (.34-1.2)	.533	.76 (.33-1.75)	.057	.33 (.10-1.03)	.226	.60 (.26-1.3)	.074	.50 (.23-1.06)	.136	.55 (.25-1.20)
	**Boot/Shoes**	.000	.05 (.01-.16)	.001	.08 (.01-.33)	.	.	.019	.16 (.03-.74)	.	.	.003	.05 (.007-.37)	.003	.11 (.02-.47)

Discussion

More than half of respondents adopted chemical method to control the pest infestation but the knowledge about the accurate rate of pest infestation was very poor. **U**se of insecticide is recommended only when pest infestation reaches to the threshold level while this study reveals respondents did not know about the actual pest infestation rate as well as they did not receive any training or skill about insecticide usage**. They** also did not use all preventing measures during the application of insecticides. Sankol *et al* 2016 also reported that 71% of farmers did not use safety measures. The education level also plays an important role. Low education level of farmers increases the risk factors in fields[6].

Data reveals most of respondents had no trainings from extension officer about the use of insecticides as Aldose *et al* (2018) 79.5% of farmers did not received any training whereas 12.3%, 5.1% and 3.1% received 2,4,and more than four trainings respectively. Similarly according to Aslam *et al* (2007) farmers of Pakistan had low level of knowledge about insecticides use however training by extension officer can be an effective step to reduce risk factors[7]. According to another study conducted in Ethiopia by Negatu *et al* (2016) 85% of the farmers had no any training regarding application of insecticides [8], while Ibitayo (2006) reported similar trend of farmers [4]. According to this study 98% of farmers did not had any training about insecticides application. In UK a study conducted regarding risk assessment about safe use of insecticides among workers. It indicated that proper training and adoption of proper safety measures can reduce the health hazards [9].

Alternative pest control method is very important component of IPM and very essential for sustainability. The result of current study shows 63% of respondents did not had any idea about alternative methods. Among 37% that know about alternative method crop rotation only practiced among farmers.

Biological control is a very effective method along with the chemical control but unfortunately due to low level of knowledge and awareness 65.3% of respondents did not know about this. This unawareness leads to force them only rely on insecticidal control method.

Discussion

Current study reported that most of the farmers were male (93.6%) while 6.4 % were female. The study conducted by Dilek oztas et al in turkey reported 100% male farmers. Similar work done by Oluewe and cheke in Nigeria 93% of farmers were male. Lekei et al also reported the same trend 93% farmers were male.

The mean age of the farmers was in range of 31-50 years. The mean age of farmers was 18-51 years reported by Tuna et al in a study done about farmer's knowledge, attitude and behavior about pesticides in Turkey. The average age of was 37.5 years according to lekei et al in Tanzania. Mubashar M et al conducted study on knowledge of farmers regarding pesticides usage and biosafety in Pakistan reported average age of farmers was 30-50 years. Similar trend was reported by Khuhru S. N et al in northern Sindh Pakistan 18-50 years.

Education level of farmers is closely related with their practices educated farmers can read and understands the instructions given on insecticides about dose preparation and precautionary measures given. The current survey showed 34% farmers were illiterate, 16% had primary level education, 8.33% had middle level education while 12.33 % were graduated from university and

11.33 % were those who get education up to higher and undergraduate level. A study conducted by Yassin, Mouraid and safi on Knowledge, attitude, practices and toxic symptoms related to use of pesticides in philistine showed 8.5% were uneducated. In nothren Sindh it was reported 12.2% were illiterate 40.9% were primary and 27.7% had middle level education. In lodhran and vehari pakistan similar trend was reported 26.4% were illiterate. These results are in Accordance with Khan and Iqbal who reported that majority of farmers in Pakistan had low level of education only 6% had university level education.

Experience of farmers is very important in order to acquire skills. This factor alone can help to increase yield and make farming cost effective. Current study represented 35% have 7-9 years of farming experience, 13% have 10-12 years while 29% had 4-5years of farming experience. The same trend was reported by Mubasher M et al 41% had 11-15 years 37.9% having 5-10 years of farming experience. Research in Pakistan represented that sociodemographic charteristics of farmers like gender, age, education level and farming experience greatly affect the use of insecticides.

Alternative pest control method is very important component of IPM and very essential for sustainability. The result of current study shows 63% of respondents did not had any idea about alternative methods. Among 37% that know about alternative method crop rotation only practiced among farmers. Biological control is a very effective method along with the chemical control but unfortunately due to low level of knowledge and awareness 65.3% of respondents did not know about this. This unawareness leads to force them only rely on insecticidal control. Khan M et al reported few farmers implement IPM and most did not know about non chemical method. According to them cultural control and mechanical methods were easy to implement. Khusro N S et al reveals 22.72% farmers practiced cultural method, 22.72% could not identify

pest and 95.45% did not use any other alternative method to control pest. The work is also supported by Yassin et al farmers did not have any knowledge about biological control or natural control agent.

Following the instruction given on the products for the preparation of proper dose is very important to prevent the overused as well as the release of residues in environment ultimately leads to development of resistance in insects. Present study reported that only 22% farmers were able to prepare the required dose while 58% farmers were unable to understand the terminologies and language of the label instructions. There was strong association between farmer's knowledge and understanding the label information of insecticides products. It leads to the higher exposure via the increase emission rate. Dalamas and Khan reported that 73% farmers were unable to read label instruction on insecticides containers. Similar trend was reported by Mubasher M et al 48.2% were unable to read instructions on insecticides label. Shetty et al reported major factor behind this is illiteracy and lack of awareness.

Failure to use of protective measures was another problem linked with the hazardous impact of insecticides on environment. A study conducted in North Greece and India reported the significant association between knowledge and use of protective measures. Clark and collegues reported the poor trend about use of protective measures among the farmers in tropics due the hot and humid climatic conditions farmers feel discomfort during use of protective measures.

The use of Protective measure become an important step for the sustainable use of insecticides likewise the IPM , irrigation method, use of less toxic agents and alternative pest control methods (use of biological agents) are the key component for sustainable agriculture. In this survey it was found that most of the farmers neglected the use of protective gears.29.7% use face mask and 3.3% use long shoes. The use of respirator was rear during spray only 1.7%

reported.40% farmers in Iran were reported to use any protective equipment during spray reported by Hashemi et al.

Furthermore improper disposal of empty containers may be another source of pesticides exposure. Mostly farmers dumped the empty insecticides containers in unsafe manners this leads to the contamination of environment due to run off, leaching and arial distribution of residues even in developing countries the practices were very common. Study in Tanzania and south Africa reported that farmers made use of empty insecticides containers for domestic purpose.

Data reveals most of respondents had no trainings from extension officer about the use of insecticides as Aldose *et al* (2018) 79.5% of farmers did not received any training whereas 12.3%, 5.1% and 3.1% received 2,4,and more than four trainings respectively. Similarly according to Aslam *et al* (2007) farmers of Pakistan had low level of knowledge about insecticides use however training by extension officer can be an effective step to reduce risk factors[7]. According to another study conducted in Ethiopia by Negatu *et al* (2016) 85% of the farmers had no any training regarding application of insecticides [8], while Ibitayo (2006) reported similar trend of farmers [4]. According to this study 98% of farmers did not had any training about insecticides application. In UK a study conducted regarding risk assessment about safe use of insecticides among workers. It indicated that proper training and adoption of proper safety measures can reduce the health hazards [9].

Conclusion

The findings of this survey demonstrate poor understanding and risky practices adopted by the farmers are the major contributing factors towards resistance development and health

issues. For underdeveloped countries like Pakistan a comprehensive and well planned program targeting on alternative pest control method and use of biological agents along with insecticides need to be initiated that can reduce the total dependency on chemicals.

The findings of this survey demonstrate poor understanding and risky practices adopted by the farmers are the major contributing factors towards resistance development and health issues. For underdeveloped countries like Pakistan a comprehensive and well planned program targeting on alternative pest control method and use of biological agents needed to be initiated that can reduce the total dependency on chemicals.

There is a dire need to start up the farmer's awareness campaigns as well as need to organize farmers by giving them trainings about proper disposal of empty containers to minimize the resistance development and resurgence of secondary pests.

There is a die need to start up the farmer's awareness campaigns as well as need to organize farmers by giving them trainings about proper disposal of empty containers to minimize the resistance development and resurgence of secondary pests.

Acknowledgement

The author sincerely thanks to the farmers and extension agriculture department for participating and contributing to this study.

Acknowledgement

The author sincerely thanks to the farmers and extension agriculture department for participating and contributing to this study. Special thanks to Dr Fayyaz Ahmed (Statistics department), University of Gujrat for his kind help in statistical analysis.

Conflict of interest

There is no conflict of interest.

References

1. Rodrigues S R A, spindola F A, Torres B J, Herbert A A S, colares F. response of different populations of seven lady beetle species to Lamda-Cyhalothrin with record of resistance. Ecotoxicology and environmental safety 2013; 96 , 53-60.
2. Abbas N, Shad S A , Ismail M, resistance to conventional and new insecticides in houseflies (diptera: muscidae) from poultry facilities in Punjab pakistan. J. Econ. Entomol.2014; 108: 826-833
3. Basit M, Saeed S, saleem MA ,Denholm I, Shah M. Detection of resistance , cross resistance and stability of resistance to new chemistry insecticides in helicoverpa armigera. J Econ Entomol 2013; 106:1414-1422.
4. Ibitayo OO, Egyptian farmers,attitude and behavior regarding aricuktural pesticides: implications for pesticides risk communication. Risk anal 2006; 26(4) 989-995.
5. Sodavy p, sitha M, Nugent r, Murphy h. situation analysis: farmers awareness and perceptions of the effects of pesticides on their health .field document.combodia :FAO Community IPM PROGRAM .2000

6. Sankoh AI, Whittle R, Semple KT, Jones KC, Sweetman AJ(2016) An assessment of the impacts of pesticide use on theenvironment and health of rice farmers in Sierra Leone.Environment International 94: 458-466.

7. Aslam M, Ali T, Zafar MI, Ahmad M (2007) Training needs offruit growers regarding pesticide use for sustainableenvironmental health in Punjab. Pakistan Journal of AgriculturalSciences 44: 511-518.

8. Negatu B, Kromhout H, Mekonnen Y, Vermeulen R (2016) Useof Chemical Pesticides in Ethiopia: A Cross-SectionalComparative Study on Knowledge Attitude and Practice ofFarmers and Farm Workers in Three Farming Systems. AnnualOccupation Hygiene 16

9. Remoundou K, Brennan M, Hart A, Frewer LJ (2014) PesticideRisk Perceptions, Knowledge, and Attitudes of Operators,Workers, and Residents: A Review of the Literature. Human and Ecological Risk Assessment 20:1113 –1138

10. Rodrigues S R A, spindola F A, Torres B J, Herbert A A S, colares F. response of different populations of seven lady beetle species to Lamda-Cyhalothrin with record of resistance. Ecotoxicology and environmental safety 2013; 96 , 53-60.

11. Abbas N, Shad S A , Ismail M, resistance to conventional and new insecticides in houseflies (diptera: muscidae) from poultry facilities in Punjab pakistan. J. Econ. Entomol.2014; 108: 826-833

12. Basit M, Saeed S, saleem MA ,Denholm I, Shah M. Detection of resistance , cross resistance and stability of resistance to new chemistry insecticides in helicoverpa armigera. J Econ Entomol 2013; 106:1414-1422.

13. Ibitayo OO, Egyptian farmers,attitude and behavior regarding aricuktural pesticides: implications for pesticides risk communication. Risk anal 2006; 26(4) 989-995.

14. Sodavy p, sitha M, Nugent r, Murphy h. situation analysis: farmers awareness and perceptions of the effects of pesticides on their health .field document.combodia :FAO Community IPM PROGRAM .2000

15. Sankoh AI, Whittle R, Semple KT, Jones KC, Sweetman AJ(2016) An assessment of the impacts of pesticide use on the environment and health of rice farmers in Sierra Leone.Environment International 94: 458-466.

16. Aslam M, Ali T, Zafar MI, Ahmad M (2007) Training needs offruit growers regarding pesticide use for sustainable environmental health in Punjab. Pakistan Journal of Agricultural Sciences 44: 511-518.

17. Negatu B, Kromhout H, Mekonnen Y, Vermeulen R (2016) Use of Chemical Pesticides in Ethiopia: A Cross-SectionalComparative Study on Knowledge Attitude and Practice of Farmers and Farm Workers in Three Farming Systems. AnnualOccupation Hygiene 16

18. Remoundou K, Brennan M, Hart A, Frewer LJ (2014) PesticideRisk Perceptions, Knowledge, and Attitudes of Operators,Workers, and Residents: A Review of the Literature. Human and Ecological Risk Assessment 20:1113 –1138

19. Khuhro, S.N., I.A. Junejo, M.H. Hullio, M.F. Hassan, S.A. Maitlo and M.A. Shaikh. 2020. Knowledge attitude practice regarding pesticide application among vegetable growers of Dadu canal irrigated areas of northern Sindh Pakistan. *Pakistan Journal of Agricultural Research*, 33(2): 331-336.

20. Khan, M., H.Z. Mahmood and C. Damalas. 2015. Pesticide use and risk perceptions among farmers in the cotton belt of Punjab, Pakistan. Crop Prot. 67: 184-190. https://doi.org/10.1016/j.cropro.2014.10.013.

21. Yassin, M.M., M.T.A. Abu and J.M. Safi. 2002. Knowledge, attitude, practice, and toxicity symptoms associated with pesticide use among farm workers in the Gaza Strip. Occup. Environ. Med., 59: 387–393. https://doi.org/10.1136/oem.59.6.387.

22. PH. Shetty, M. Murugan, MB. Hiremath, and KG. Sreeja, "Farmerss education and perception on pesticide use and crop economies," in *in Indian agriculture. JES*, vol. 1, p. 3, 3-8, 1(1, 2010.

23. S. H. Hashemi, S. M. Hosseini, and M. K. Hashemi, "Farmer's perceptions of safe use of pesticides: Determinants and training needs," *International Archives of Occupational and Environmental Health*, vol. 85, pp. 57–66, 2012.

9.

Formatted: Font: (Default) Times New Roman, 12 pt

Formatted: Normal, No bullets or numbering

RESPONSE TO REVIEWER

Reviewer	Comment	Response
1	This section is too short and did not cover the state of knowledge for the last ten years. It should be enlarged and surveyed the current state of the arts for at least for the past 10 years .	Introduction is modified recent work references added.
	Add detailed description on the questionnaire .	Description added.<br>A structured questionnaire having open ended and close ended questions was used to collect information by using Urdu and Punjabi Language. The questionnaire consist up of three sections, First part is related to sociodemographic characters of farmers such as Gender, marital status, Age, Education level , Farm area, irrigation method, farming Experience and working hours they spend in their crops were determined. Second part related to perceptions like which insecticide is better and they prefer to use more, which crop and which pest most frequently targeted, how they prepare the spray dose for targeted pests, they have any idea about alternate pest control method like IPM, Biological agent or natural enemies and third part related to the attitude and practices of farmers like how hey protect themselves during spray, they use protective equipment or not and they acquired any skill or training from any.
	was is face to face interview	yes it is face to face conversation
	where are the statistical analysis of the work	Detail of statistical work added.
	The authors used 300 farmers as a sample size but they did not justify it and/or how they collected it.<br>The methodology of selection is not clearly described. the inclusion and exclusion criteria is not	Detail of this added.<br>A cross sectional study was conducted during October 2019 to February 2020 by using questionnaire based interview of farmers. In this survey face to face conversation was done with farmers. Firstly in local language aim of study was described later on after their willingness questions were

	mentioned	asked verbally and their response was noted on questionnaire. This survey was carried out in industrial triangle including Gujrat, Gujranwala and Sialkot. These sites were selected on the basis of operational convenience and industrial importance of this region. Due to availability of water and suitability of climatic conditions various crops like wheat, rice, fruits and vegetables cultivated in this region. Farmers of this region use insecticides abundantly in this region to protect their crops from pests.<br><br>In first step questionnaire was prepared and survey was conducted from a sub sample of 20 Farmers who were reluctant to participate in study. On the basis of results of pretest this survey was further modified to reduce the deficiencies felt at that time. In second step a comprehensive survey was conducted among 300 farmers of District Gujrat, Gujranwala and Sialkot of Punjab province.
	what was the response rate, was any objection to participate in the study, if yes, add the rate	A total of 300 farmers from three Districts were participated in the study. Random sampling was done. Most of the farmers were involved in cultivation of wheat, rice, millet, sorghum and vegetables in this region. The response rate was 100% farmers did not refuse to participate.
	add scientific names to all pests	Family name added of each pest.<br>*Ustilaginaceae*<br>*Aphidoidea*<br>*Aleyrodidae*<br>*Amrasca biguttula*<br>*Golovinomyces orontii*<br>*Pseudococcidae*<br>*Isoptera*
	specify; high volume or low volume	
	add definition<br>**cultural control**	cultural control refers to the manipulation of old practices like date management with respect to pest outbreak

	Sum of percentage should be 100. what about the missing data, how did they treat containers	Changes done.<br>These farmers 50.33 % burry the empty containers and 14% of respondents burnt them while 31.67% throw them in trash without any processing while 3.67% of farmers go for Government collection centers for disposal of empty containers of insecticides
	Discussion - this section is rather weak and should be rewritten. author should discuss his work with previous published works<br><br>El-Nahhal, Y. (2017) Risk Factors among Greenhouse Farmers in Gaza Strip. Occupational Diseases and Environmental Medicine, 5, 1-10. doi: 10.4236/odem.2017.51001.<br><br>Nahhal, Y. (2016) Biochemical Changes Associated with Long Term Exposure to Pesticide among Farmers in the Gaza Strip. Occupational Diseases and Environmental Medicine, 4, 72-82. doi: 10.4236/odem.2016.43009.<br><br>Knowledge, attitude, practice, and toxicity symptoms associated with pesticide use among farm workers in the Gaza Strip<br>MM Yassin, TAA Mourad, JM Safi<br>Occupational and environmental medicine 59 (6), 387-393	**Discussion**<br>rewritten
	References- this section	Updated.

	should be updated	
**Reviewer 2**		
	- The introduction is inadequate and should be expand it. The detailed aim should be given at last paragraph of introduction.	Introduction section modified, expanded and detail added.
	-The reasons for the preference of three sub-regions should be explained and if the differences there are should be given in the text such as crop, irrigation, topographic, climate, etc.	A cross sectional study was conducted during October 2019 to February 2020 by using questionnaire based interview of farmers. In this survey face to face conversation was done with farmers. Firstly in local language aim of study was described later on after their willingness questions were asked verbally and their response was noted on questionnaire. This survey was carried out in industrial triangle including Gujrat, Gujranwala and Sialkot. These sites were selected on the basis of operational convenience and industrial importance of this region. Due to availability of water and suitability of climatic conditions various crops like wheat, rice, fruits and vegetables cultivated in this region. Farmers of this region use insecticides abundantly in this region to protect their crops from pests.
	-The abstract mentioned Anova analyses and the result has the univariate regression model. However, the analysis method is not in the material and method. Please give a detailed analysis method.	Detailed analysis method is added now.
	There are no statistical results and these decreases its value for publication in this journal.	Statistical analysis along with results added now.
	-I did not understand the terminology (%age) in your tables. You mean percentage, please change it.	% age replaced with percentage.
	- Discussion is inadequate please expand it. You have three sub-region, you can	Discussion is expanded now.

	compare them.		
	Reference should be rearranged according to journal guidelines	Changes done according to journal guideline.	
