# Supplementary material for: A cross sectional survey of knowledge, attitude and practices related to the use of insecticides among farmers in industrial triangle of Punjab, Pakistan
Source: PLoS One. 2021 Aug 19;16(8):e0255454. doi: 10.1371/journal.pone.0255454 (PMC8376108; doi:10.1371/journal.pone.0255454)
Supplement: Attachment — Submitted filename: RESPONSE TO REVIEWER.docx [file pone.0255454.s005.docx]

**RESPONSE TO REVIEWER**

| **Reviewer** | **Comment** | **Response** |  |
| --- | --- | --- | --- |
| **1** | This section is too short and did not cover the state of knowledge for the last ten years. It should be enlarged and surveyed the current state of the arts for at least for the past 10 years . | Introduction is modified recent work references added. |  |
|  | Add detailed description on the questionnaire . | Description added.  A structured questionnaire having open ended and close ended questions was used to collect information by using Urdu and Punjabi Language. The questionnaire consist up of three sections, First part is related to sociodemographic characters of farmers such as Gender, marital status, Age, Education level , Farm area, irrigation method, farming Experience and working hours they spend in their crops were determined. Second part related to perceptions like which insecticide is better and they prefer to use more, which crop and which pest most frequently targeted, how they prepare the spray dose for targeted pests, they have any idea about alternate pest control method like IPM, Biological agent or natural enemies and third part related to the attitude and practices of farmers like how hey protect themselves during spray, they use protective equipment or not and they acquired any skill or training from any. |  |
|  | was is face to face interview | yes it is face to face conversation |  |
|  | where are the statistical analysis of the work | Detail of statistical work added. |  |
|  | The authors used 300 farmers as a sample size but they did not justify it and/or how they collected it.  The methodology of selection is not clearly described. the inclusion and exclusion criteria is not mentioned | Detail of this added.  A cross sectional study was conducted during October 2019 to February 2020 by using questionnaire based interview of farmers. In this survey face to face conversation was done with farmers. Firstly in local language aim of study was described later on after their willingness questions were asked verbally and their response was noted on questionnaire. This survey was carried out in industrial triangle including Gujrat, Gujranwala and Sialkot. These sites were selected on the basis of operational convenience and industrial importance of this region. Due to availability of water and suitability of climatic conditions various crops like wheat, rice, fruits and vegetables cultivated in this region. Farmers of this region use insecticides abundantly in this region to protect their crops from pests.  In first step questionnaire was prepared and survey was conducted from a sub sample of 20 Farmers who were reluctant to participate in study. On the basis of results of pretest this survey was further modified to reduce the deficiencies felt at that time. In second step a comprehensive survey was conducted among 300 farmers of District Gujrat, Gujranwala and Sialkot of Punjab province. |  |
|  | what was the response rate, was any objection to participate in the study, if yes, add the rate | A total of 300 farmers from three Districts were participated in the study. Random sampling was done. Most of the farmers were involved in cultivation of wheat, rice, millet, sorghum and vegetables in this region. The response rate was 100% farmers did not refuse to participate. |  |
|  | add scientific names to all pests | Family name added of each pest.  *Ustilaginaceae*  *Aphidoidea*  *Aleyrodidae*  *Amrasca biguttula*  *Golovinomyces orontii*  *Pseudococcidae*  *Isoptera* |  |
|  | specify; high volume or low volume |  |  |
|  | add definition  **cultural control** | cultural control refers to the manipulation of old practices like date management with respect to pest outbreak |  |
|  | Sum of percentage should be 100. what about the missing data, how did they treat containers | Changes done.  These farmers 50.33 % burry the empty containers and 14% of respondents burnt them while 31.67% throw them in trash without any processing while 3.67% of farmers go for Government collection centers for disposal of empty containers of insecticides |  |
|  | Discussion - this section is rather weak and should be rewritten. author should discuss his work with previous published works  El-Nahhal, Y. (2017) Risk Factors among Greenhouse Farmers in Gaza Strip. Occupational Diseases and Environmental Medicine, 5, 1-10. doi: 10.4236/odem.2017.51001.  Nahhal, Y. (2016) Biochemical Changes Associated with Long Term Exposure to Pesticide among Farmers in the Gaza Strip. Occupational Diseases and Environmental Medicine, 4, 72-82. doi: 10.4236/odem.2016.43009.  Knowledge, attitude, practice, and toxicity symptoms associated with pesticide use among farm workers in the Gaza Strip  MM Yassin, TAA Mourad, JM Safi  Occupational and environmental medicine 59 (6), 387-393 | **Discussion**  rewritten |  |
|  | References- this section should be updated | Updated. |  |
| **Reviewer 2** |  |  |  |
|  | - The introduction is inadequate and should be expand it. The detailed aim should be given at last paragraph of introduction. | Introduction section modified, expanded and detail added. |  |
|  | -The reasons for the preference of three sub-regions should be explained and if the differences there are should be given in the text such as crop, irrigation, topographic, climate, etc. | A cross sectional study was conducted during October 2019 to February 2020 by using questionnaire based interview of farmers. In this survey face to face conversation was done with farmers. Firstly in local language aim of study was described later on after their willingness questions were asked verbally and their response was noted on questionnaire. This survey was carried out in industrial triangle including Gujrat, Gujranwala and Sialkot. These sites were selected on the basis of operational convenience and industrial importance of this region. Due to availability of water and suitability of climatic conditions various crops like wheat, rice, fruits and vegetables cultivated in this region. Farmers of this region use insecticides abundantly in this region to protect their crops from pests. |  |
|  | -The abstract mentioned Anova analyses and the result has the univariate regression model. However, the analysis method is not in the material and method. Please give a detailed analysis method. | Detailed analysis method is added now. |  |
|  | There are no statistical results and these decreases its value for publication in this journal. | Statistical analysis along with results added now. |  |
|  | -I did not understand the terminology (%age) in your tables. You mean percentage, please change it. | % age replaced with percentage. |  |
|  | - Discussion is inadequate please expand it. You have three sub-region, you can compare them. | Discussion is expanded now. |  |
|  | Reference should be rearranged according to journal guidelines | Changes done according to journal guideline. |  |
|  |  |  |  |
