# Supplementary material for: A cross sectional survey of knowledge, attitude and practices related to the use of insecticides among farmers in industrial triangle of Punjab, Pakistan
Source: PLoS One. 2021 Aug 19;16(8):e0255454. doi: 10.1371/journal.pone.0255454 (PMC8376108; doi:10.1371/journal.pone.0255454)
Supplement: Attachment — Submitted filename: RESPONSE TO REVIEWER.docx [file pone.0255454.s007.docx]

**RESPONSE TO REVIEWER**

| **Reviewer** | **Comment** | **Response** |
| --- | --- | --- |
|  | Abstract  Grammatical mistake and spelling mistakes. | The Abstract has been revised as per suggested comments . e.g missing word “use” is now incorporated and use of comma has been omitted accordingly |
|  | Introduction  In this portion punctuation marks, grammar and spelling mistakes were highlighted.  At few point there was mistake of use of capital letters. | In this portion all mistakes of comma, punctuation, sentence structure, grammar and spelling are addressed and correction has been done at level best. |
|  | Interview procedure  In this portion spelling and punctuation mistake were reported. | All possible correction has been incorporated. |
|  | Knowledge of respondents  This does not refer to knowledge, this part talks more about characteristic of respondents, the description of group of respondents. | This heading is modified as “Description of respondents” |
|  | Table 02  To my opinion, this data would be much nicer if they would be presented graphically. | All data in Table 02 is now represented in the form of graphs. |
|  | Knowledge of respondents regarding pesticides  In this portion spelling and grammar mistakes were mentioned | Correction of Spelling mistakes and grammar mistakes has been addressed. |
|  | Common practices of farmers  I suggest to use only the term farmers or only the term respondents through the whole manuscript. | Term respondents instead of farmers is now used in whole manuscript. |
|  | This paragraph should be a new separate subchapter Statistical analysis. | Subchapter “Statistical analysis” added |
|  | Table 05  Here I do not understand well the table.  Column refers to the education level of farmers - but which column to which education level? All columns are just described as OR (95 % CI).  What does it mean "B8a"? | Table 05 is now modified according to the given instructions.  In statistical analysis when data was put on SPSS this question was coded as B8a by mistake it was placed here now correction has been incorporated accordingly |
|  | Discussion   - Format style of et al - Year of publication - Capitalization of letters | Correction has been employed accordingly |
|  | References- this section should be updated | Said section has been modified accordingly |
|  | Caption of figures | Correction done  In main manuscript the captions of figures placed at their respective places. |
